# Numerical Approaches for Recovering the Deformable Membrane Profile of Electrostatic Microdevices for Biomedical Applications

**DOI:** 10.3390/s23031688

**Published:** 2023-02-03

**Authors:** Mario Versaci, Francesco Carlo Morabito

**Affiliations:** DICEAM Department, “Mediterranea” University, 89124 Reggio Calabria, Italy

**Keywords:** electrostatic membrane micropumps, electrostatic fringing field, 2D circular steady-state semi-linear elliptic models, numerical ghost solutions, pull-in voltage

## Abstract

Recently, a circular symmetrical nonlinear stationary 2D differential model for biomedical micropumps, where the amplitude of the electrostatic field is locally proportional to the curvature of the membrane, was studied in detail. Starting from this, in this work, we first introduce a positive and limited function to model the dielectric properties of the material constituting the membrane according to experimental evidence which highlights that electrostatic capacitance variation occurs when the membrane deforms. Therefore, we present and discuss algebraic conditions of existence, uniqueness, and stability, even with the fringing field formulated according to the Pelesko–Driskoll theory, which is known to take these effects into account with terms characterized by reduced computational loads. These conditions, using “gold standard” numerical approaches, allow the optimal numerical recovery of the membrane profile to be achieved under different load conditions and also provide an important criterion for choosing the intended use of the device starting from the choice of the material constituting the membrane and vice versa. Finally, important insights are discussed regarding the pull-in voltage and electrostatic pressure.

## 1. Introduction

As known, microelectromechanical systems (MEMS) allow for the creation of both sensors and actuators capable of using non-electrical devices in microchips [1,2]. Fluidic devices such as micropumps were historically the first to be made and, with the discovery of new pharmacological therapies [3,4,5,6], today they represent a highly developed research area. Currently, microfluidics is based on sophisticated physical-mathematical models which, unlike macroscopic applications, are able to take into account electrokinetic [7], magnetohydrodynamic [8], electrochemical [9], electrostatic [1] and other effects [10,11]. Concerning the design of a micropump, the actuator is one of the most important devices since it converts the applied external energy (electrical, thermal, …) into movement of the deformable element (usually, a membrane). The mechanical micropumps (piezoelectric [12], electrostatic [13], thermopneumatic [14], electromagnetic [15], bimetallic [16], ion conductive [17], phase change [18], shape memory alloy polymer films [19]) require an actuator for pumping; usually, they consist of a pumping chamber separated by a deformable diaphragm so the fluid flows by means of oscillations which generate pressures depending on the volume of the stroke inside the chamber produced by the actuator. Of course, performance is severely limited by the mechanical components. Non-mechanical micropumps, having no moving parts, do not require mechanisms for converting the energy administered into kinetic momentum. However, while easy to design and build, they can pump low conductivity fluids [20,21,22,23,24,25,26]. Research efforts are currently focused on the efficiency of drug administration to be combined with the efficiency of the fluidic device and of the micropumps (implantable and/or removable) based on MEMS technology since positive results in various clinical conditions depend on them [27,28]. Since the release and absorption of drugs strongly depends on pharmacokinetic and metabolic mechanisms, in order to reduce adverse phenomena, research is oriented towards the development of devices capable of controlling the dosage rate from the delivery system [29,30]. MEMS-based micropumps are designed to deliver drugs at a specific rate in accordance with the required pharmacokinetics also allowing prolonged infusion volume control (useful for certain pathologies) and improving the patient’s quality of life [31,32,33,34]. Electrostatic membrane MEMS micropumps currently have strategic importance as they offer a high level of performance with almost a total absence of maintenance interventions [28,35,36]. Furthermore, their easy design as well as their low cost in the manufacturing step allow for large-scale industrial production [37,38].

Although the production of electrostatic membrane MEMS micropumps has reached considerable levels, there is still a need to study new and accurate physical-mathematical models to test, in the design phase, the performance of the micropumps stressed by the different types of loads to which they will be subjected during their use. Recently, new models for electrostatic membrane micropumps based on integro-differential equations have been presented, whose solutions, also depending on the charge densities, can be obtained by analytical procedures [39,40]. Even if such models realistically simulate the multi-physics present in the devices, the high computational loads they entail do not facilitate hardware prototyping. Therefore, the development of models (especially those with a high degree of symmetry) with a reduced computational load will allow hardware to operate at a lower cost [41,42,43,44,45,46,47].

Concerning circular steady-state electrostatic membrane MEMS micropumps, recently, a dimensionless second-order semilinear elliptic model with singularity (whose solution was the profile of the deformable membrane, u(r), r∈[0,R], with *r* being the radial coordinate and *R* being the radius of the micropump membrane) was studied in [48,49,50], without considering effects due to the fringing field, where important results of existence [48] and uniqueness were obtained [49] without, however, guaranteeing any stability [50]. In particular,

(1) $$\left\{\begin{array}{c} \frac{d^{2}u\left(r\right)}{dr^{2}}=-r^{-1}\frac{du(r)}{dr}-{\left(\theta \lambda \right)}^{-1}{\left(1-u\left(r\right)-d^{*}\right)}^{2}\\ u\left(R\right)=0,\;\;\;\;\frac{du(0)}{dr}=0,\;\;\;0<u\left(r\right)<d. \end{array}\right.$$

where $d\in R^{+}$ is the distance between the membrane at rest and the counter-electrode. λ, as defined in (4), is a positive dimensionless parameter that depends on both the applied voltage, *V*, and the mechanical tension, *T*, of the membrane at rest. $d^{*}$ is the critical security distance that ensures that the membrane does not touch the counter-electrode. $\theta \in R^{+}$ (physical parameter without limitation) considers the electrical properties of the membrane [51,52], so that θλ, which is subjected to any limitation if numerical recovery of the membrane is performed, expresses the electro-mechanical properties of the membrane.

Model (1) is interesting because it models a device that is controllable in terms of voltage because, as shown in (4), λ depends on *V* (so that λ, like *V*, is a bounded parameter). Moreover, it does not consider the effects due to fringing [53,54], and in parallel, it is not particularly adherent to the main requirements of micropumps for drug delivery systems because the dielectric properties of the membrane are modelled by a function, f(r)=1, ∀r∈[0,R], as if the membrane, dielectrically exhibits the same behavior at each of its points. In the recent past, important results have been obtained concerning analogous models of MEMS devices, even in the presence of fringing field, without defining a suitable dielectric profile of the membrane [51], while the dielectric profile was formulated according to some experimental evidences, where, however, the deformable element was a rectangular metal plate [52]. Finally, any instability of the membrane could cause electrostatic discharge. It is worth noting that, when the membrane deforms, strong variation in the electrostatic capacitance occurs in the device, for which an auxiliary electrostatic capacitance, $C_{f}$, is needed to oppose the variation of *V*.

With these premises, the main purpose of this work can be summarized by the following points:

1. As in [51], to take into account the effects due to the fringing field (usually dependent on the diameter/height ratio of the device that can increase the risk of electrostatic discharge being generated by possible contact of the membrane with the couter-electrode), a weighted addend has been considered in (1) according to the Pelesko–Driskoll theory [55], achieving a 2D second-order nonlinear differential model with singularity and circular symmetry.
2. The proposed model, also obtained by considering that the straight line of the electric field, E, on the membrane is locally proportional to the curvature of the membrane itself, models the behavior of the micropump as a function of the electromechanical and dielectric properties of the material constituting the membrane. Therefore, a positive bounded function, f(r), ∀r∈[0,R], is introduced to simulate, according to the experimental results known in the literature, the dielectric properties of the membrane to accommodate the electrostatic capacitance changes that occur as the membrane deforms, while the electromechanical properties are formalized by the product θλ that is already present in (1).
3. The proposed model is formulated in order for known remarkable results to be exploited to prove the existence, uniqueness, and stability (in relation to *V* applied) of the solution by providing an algebraic condition depending on f(r). If this is satisfied by the numerical solutions obtained, the profiles of the recovered membrane do not represent ghost solutions (i.e., numerical solutions that do not satisfy the conditions required for the existence, uniqueness, and stability of the analytical solution).
4. Furthermore, after some calculations and considerations, an important limitation concerning the total electrostatic force (also considering the contribution due to the fringing field) in the micropump is achieved depending on both *V* and *T*, so as to connect this force with both the intended use of the device and the choice of material constituting the membrane.
5. Specific numerical techniques considered the “gold standard” for these kinds of problems, characterized by different levels of convergence for showing efficiency and performance, were implemented in MatLab^®^ R2022 running on Intel Core 2 CPU at 1.45 GHz to perform the numerical recovery of the membrane, providing results that are compatible with the existence and uniqueness conditions required for the solution. These results are all related to both *V* and *T*, obtaining a criterion that can be used for choosing a graphical approach on the Cartesian plane (V,T). Furthermore, the minimum value of *V* necessary to overcome the inertia of the membrane in the startup phase was quantified.
6. Moreover, the pull-in voltage and the electrostatic pressure depending on both *V* and *T* were quantified so that these values are immediately attributable to the choice of membrane material and the intended use of the micropump.

The remainder of the work is organized as follows: Section 2 details the studied micropump, framing the problem from the points of view of both the actuator and the transducer. Then, Section 3 describes the proposed physical-mathematical model, presenting important results related to the existence, uniqueness, and stability of the solutions (in Section 4, Section 5, Section 6 and Section 7). After the dielectric properties of the membrane have been modeled, an important limitation for the electrostatic force is presented and discussed (Section 8). Next, Section 9 is dedicated to the numerical recovery of the membrane profile for both different loading conditions and different effects due to the fringing field. Then, after quantifying the *V* needed to overcome the mechanical inertia of the membrane (Section 10), a link between *V* and *T* is proposed in Section 11. Finally, before concluding the paper, possible future developments are provided (Section 13), and some considerations regarding both the pull-in voltage and electrostatic pressure are discussed in Section 12.

## 2. The Electrostatic Membrane Micropump

### 2.1. Electrostatic Membrane Micropump as an Actuator

**Figure 1:**
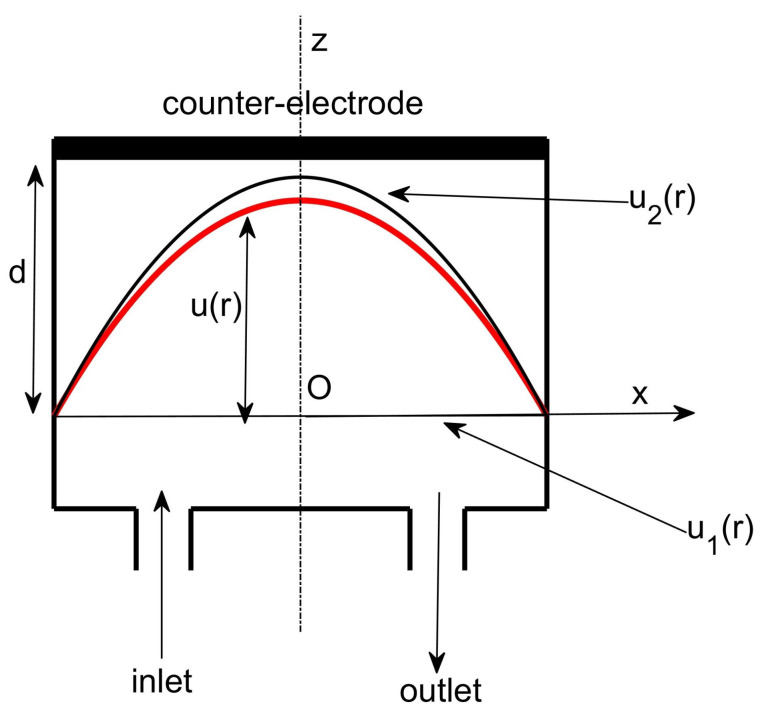
The micropump: u(r) vs. *r*: visualization of u(r), 1−u(r), $d^{*}$ and the upper and lower solutions $u_{1}\left(r\right)$ and $u_{2}\left(r\right)$.

Let us consider the usual 3D Euclidean space, $R^{3}$, in which a system of orthonormal Cartesian axes Oxyz (*z*, vertical axis) is present. The studied micropump, displayed in Figure 1, consists of two parallel circular plates of radius *R* arranged at a distance *d* from each other; the lower plate, on the xy plane in order with its center located on r=0, is at zero potential, while the upper one is at *V* potential. A circular membrane of the same radius is anchored to the edges of the lower plate, which, under the action of *V* (and therefore of the electric field E which is established between the plates), deforms towards the counter-electrode (top-plate) due to the electrostatic pressure, $p_{el}$. During the deformation of the membrane, the volume of the chamber below it is modified by withdrawing the drug from the inlet check valve. Once the action of *V* is cancelled, E cancels itself and the membrane returns to its resting state with consequent expulsion of the liquid from the outlet valve. To overcome the mechanical inertia of the membrane without the fringing field, *V* must establish an electrostatic field E inside the micropump that is capable of generating an electrostatic pressure, $p_{el}\geq 0.5\epsilon_{0}{\left|E\right|}^{2}$ ($\epsilon_{0}$, permittivity of the free space), by imparting to the membrane an electrostatic force, $f_{el}$ that is equal to at least $0.5\epsilon \pi R^{2}V^{2}{\left(d-u\left(r\right)-d^{*}\right)}^{-2}$, obtaining a displacement in the center [51,53],

(2) $$u_{0}=R^{2}p_{el}/4T.$$

Here, we used $\pi R^{2}$ to formulate the surface area of the membrane (even if the membrane deforms under the effect of the applied external electric voltage). This makes sense because d≪R; hence, the area of the strained membrane is approximately equal to the area of the membrane at rest. As the membrane deforms, it makes E (dependent on d−u(r)), so the capacitance of the device $C_{el}$ is variable) locally orthogonal to the tangent line to the membrane. Furthermore, the curvature of the membrane, K(r,u(r)) [51], depends on |R|, so [50,51,52]

(3) $$\left|E\right|\propto K\left(r,u\left(r\right)\right)=0.5\left(\frac{d^{2}u\left(r\right)}{dr^{2}}+r^{-1}\frac{du(r)}{dr}\right).$$

It should be noted that the width, *L*, of the studied micropump is such that 2R≪d due to the effects attributable to the fringing field should not be neglected. Moreover, [50,51,52,53],

(4) $$\lambda ={\left(d^{3}T\right)}^{-1}\epsilon_{0}V^{2}\left(2R^{2}\right)<\lambda^{*},$$

where $\lambda^{*}$ represents the pull-in voltage in order for no bifurcation phenomena to occur. In other words, $\lambda \in L^{\infty }$ and ${\left||\lambda |\right|}_{\infty }<\lambda^{*}$.

### 2.2. Electrostatic Membrane Micropump as a Transducer

From the theory of membrane mechanics, if *p* is the mechanical pressure, the profile u(r) can be obtained from [48,49,50,53]

(5) $$u\left(r\right)=u_{0}{\left(1-{\left(r^{-1}R\right)}^{2}\right)}^{2}p,$$

where $u_{0}=pR^{4}/64D$, with *D*, the stiffness (here considered as a coefficient), so that the device works as a transducer achieving [48,49,50,53]

(6) $$C_{el}\left(u_{0}\right)=\int_{0}^{R}2\epsilon_{0}\pi r{\left(d\left(1-d^{-1}u\left(r\right)\right)\right)}^{-1}dr,\;\;\;\mathrm{with}\;\;\left|u_{0}\right|\ll d.$$

Moreover, exploiting the Taylor series up to the third term, from (6), we can write

(7) $$C_{el}\left(u_{0}\right)=C_{el}\left(0\right)+C_{el}^{\prime }\left(0\right)u_{0}+0.5C_{el}^{\prime \prime }\left(0\right)u_{0}^{2}.$$

Being

(8) $$C_{el}\left(0\right)=\int_{0}^{R}2\epsilon_{0}\pi rd^{-1}dr=\epsilon_{0}\pi R^{2}d^{-1}=C_{0},$$

(9) $$C_{el}^{\prime }\left(u\right)=2\epsilon_{0}\pi Rd^{-1}\left(1-ud^{-1}\right)$$

so that

(10) $$C_{el}^{\prime }\left(0\right)=2\epsilon_{0}\pi Rd^{-1},$$

(11) $$C_{el}^{\prime \prime }\left(u\right)=2\epsilon_{0}\pi Rd^{-2}{\left(1-ud^{-1}\right)}^{-2}$$

and

(12) $$C_{el}^{\prime \prime }\left(0\right)=2\epsilon_{0}\pi Rd^{-2};$$

therefore, setting R≈6d (as many industrial production processes suggest), (7) becomes

(13) $$C_{el}\left(u_{0}\right)\approx C_{0}\left(1+3u_{0}d^{-1}+6u_{0}^{2}d^{-2}\right),$$

where $C_{0}=\epsilon \pi r^{2}d^{-1}$, from which the electrostatic charge of the membrane, the co-energy of the system, and the electrostatic force are obtainable together with |E(r)|, which can be written as

(14) $$\left|E\left(r\right)\right|\approx V{\left(d-u_{0}{\left(1-{\left(rR^{-1}\right)}^{2}\right)}^{2}\right)}^{-1}.$$

Obviously, in this case, being the deformable element a metallic plate, *D* assumes a significant value generating a strong limitation of u(r) which affects the fact that d−u(r) is replaceable by *d* (because u(r) can be considered negligible). However, in our problem, the deformable element is a membrane so that *D* is negligible, and $u_{0}$ also increases, increasing the risk that the membrane will touch the counter-electrode. In this case [53]:

(15) $$u\left(r\right)=u_{0}\left(1-{\left(rR^{-1}\right)}^{2}\right)$$

where u=0 is formulated as shown in (2).

### 2.3. The Transducer as an Understanding Device of the Actuator

As already highlighted in [48,49,50,53], *p* and $p_{el}$ exhibit a formalizable link, since E, produced by *V*, generates $p_{el}$, pushing the membrane towards the counter-electrode. Then, from (2), it is easy to achieve

(16) $$u_{0}=kp_{el}.$$

where *k* is an important parameter that governs the existence and uniqueness of the solution for the proposed model with the fringing field as specified in the following sections.

## 3. The Proposed Model

In (1), $\lambda /{\left(1-u\left(r\right)-d^{*}\right)}^{2}$ represents a term proportional to |E| [50,51,53]. As anticipated above, we introduce a positive bounded function, f(r), to define the dielectric properties of the membrane (usually referred to in the literature as the dielectric profile of the membrane). Furthermore, to take into account the effects due to the fringing field, we consider an additional term that is equal, according to the Pelesko–Driscoll approach, to ${\lambda f\left(r\right)\delta |\nabla u\left(r\right)|}^{2}$ [51], where $\delta \in R^{+}$ weighs the effects due to the fringing field. Then, (1) becomes

(17) $$\left\{\begin{array}{c} \Delta u\left(r\right)=-{\left(1-u\left(r\right)-d^{*}\right)}^{-2}{\lambda (1+\delta |\nabla u\left(r\right)|}^{2})\\ u\left(R\right)=0,\;\;\;\;\frac{du(0)}{dr}=0,\;\;\;0<u\left(r\right)<d. \end{array}\right.$$

Furthermore, by exploiting the radial symmetry with respect to the vertical axis and considering that $\nabla u\left(r\right)=\frac{du(r)}{dr}$, (17) can be written as

(18) $$\left\{\begin{array}{c} r^{-1}\frac{du(r)}{dr}+\frac{d^{2}u\left(r\right)}{dr^{2}}=-{\left(1-u\left(r\right)-d^{*}\right)}^{-2}\lambda \left(1+\delta |\frac{du(r)}{dr}{|}^{2}\right)\\ u\left(R\right)=0,\;\;\;\;\frac{du(0)}{dr}=0,\;\;\;0<u\left(r\right)<d. \end{array}\right.$$

Moreover, by exploiting the idea that ${\left|E\right|}^{2}\propto \lambda f\left(r\right){\left(1-u\left(r\right)-d^{*}\right)}^{-2}$, it makes sense to write

(19) $$\lambda f\left(r\right){\left(1-u\left(r\right)-d^{*}\right)}^{-2}=\theta {\left|E\right|}^{2},\;\;\;\;\;\theta \in R^{+}.$$

In the past, it was proven that E is locally orthogonally to the straight-line tangent to the membrane [50,51,53]. Therefore, considering K(r,u(r)) as the curvature of the membrane (see (3)), we can write |E|∝K(r,u(r)), whose function of proportionality $\mu \left(r,u\left(r\right),\lambda ,f\left(r\right)\right)=\lambda f\left(r\right){\left(1-u\left(r\right)-d^{*}\right)}^{-1}$ belongs to $C^{0}\left(\left[-0.5,0.5\right]\times \left[0,1\right)\times \left[0,1\right]\times \left[0,1\right]\right)$ which in [51] has been shown to have no limitation. Therefore, (18), after some calculations, becomes

(20) $$\left\{\begin{array}{c} \frac{d^{2}u\left(r\right)}{dr^{2}}+r^{-1}\frac{du(r)}{dr}=-4{\left(1-u\left(r\right)-d^{*}\right)}^{-2}\theta \lambda f\left(r\right){\left(\frac{d^{2}u\left(r\right)}{dr^{2}}+r^{-1}\frac{du(r)}{dr}\right)}^{2}\left(1+\delta |\frac{du(r)}{dr}{|}^{2}\right)\\ u\left(R\right)=0,\;\;\;\;\frac{du(0)}{dr}=0,\;\;\;\theta \in R^{+},\;\;\;\;0<u\left(r\right)<d, \end{array}\right.$$

where

(21) $$4{\left(1-u\left(r\right)-d^{*}\right)}^{-2}\theta \lambda f\left(r\right),$$

represents the electromechanical load on the membrane when *V* is applied (*V* generates E between the electrodes which determines $f_{el}$ which, for each surface unit, is transformed into $p_{el}$. Obviously, model (20) is difficult to solve analytically; therefore, in the first instance, we verified the existence, uniqueness, and stability of the solution in this new formulation where f(r) takes place. It is worth noting that, in (20), local mechanical stresses were not considered in order not to make the model depend on tensor variables which would further reduce $u_{0}$. However, not considering this contribution allows for a possible overestimated numerical recovery “with a safety advantage”, since there is the certainty that the real value of $u_{0}$ is lower than the numerically obtained one. Thus, the membrane will certainly not touch the counter-electrode of the micropump. We also observed that f(r), which influences the electrostatic charge in the micropump, is decisive for the functioning of the micropump, especially when the deformation of the membrane is the maximum allowed, as already experimented in similar devices [56,57]. This is due to the fact that $u\left(r\right)<1-d^{*}$ because, physically, the membrane must not touch the top plate (mathematically imposed in (20)). Then, if $u\left(r\right)=1-d^{*}$, from (20), it follows that f(r)=0, so (21) will vanish.

Furthermore, (20) models circular electrostatic micropumps, even for small displacements of the membrane. Indeed, if $\pi R^{2}\gg d^{2}$, δ will be negligible. However, from $\pi R^{2}\gg d^{2}>u^{2}\left(r\right)$, it follows that $u\left(r\right)\ll \sqrt{\pi R^{2}}\approx 10^{-6}$ m, which provides the possibility of performing small displacements of the membrane. Obviously, (20) is formulated with an extremely simplified micropump geometry, allowing a preliminary analytical study to be carried out, of which the results, still unconformed by experimentation, provide an important theoretical contribution.

**Remark** **1.** *Model* (1) *is a simplification of the following model*

(22) $$\rho h\frac{\partial^{2}w^{\prime }}{\partial t^{\prime 2}}+a\frac{\partial w^{\prime }}{\partial t^{\prime }}-T\nabla^{2}w^{\prime }+D\Delta^{2}w^{\prime }=-0.5\epsilon_{0}{\left|\nabla \phi \right|}^{2}$$

*where ρ is density and h is the thickness of the membrane. Defining*

(23) $$u=\frac{w^{\prime }}{d},\;\;\Phi =\frac{\phi }{V},\;\;\;x=\frac{x^{\prime }}{L},\;\;\;y=\frac{y^{\prime }}{L},\;\;\;z=\frac{z^{\prime }}{d},\;\;\;t=\frac{Tt^{\prime }}{aL^{2}},$$

*it is easy to achieve*

(24) $$\left\{\begin{array}{c} \epsilon^{2}\Delta \Phi +\frac{\partial^{2}\Phi }{\partial z^{2}}=0\\ \frac{1}{\alpha^{2}}\frac{\partial^{2}u}{\partial t^{2}}+\frac{\partial u}{\partial t}-\Delta u+\zeta \Delta^{2}u=-\lambda {\left(\epsilon^{2}{\left|\nabla \phi \right|}^{2}+\frac{\partial \phi }{\partial z}\right)}^{2}) \end{array}\right.$$

*where Φ=1 on the membrane and Φ=0 on the support plate. Moreover,*

(25) $$\alpha =\frac{aL}{\sqrt{\rho hT}},\;\;\;\zeta =\frac{D}{TL^{2}},\;\;\;\epsilon =\frac{d}{L}$$

*and λ is formulated as in* (4). (24) *represents a system of nonlinear coupled partial differential equations which hardly provides exact solutions even for extremely simplified geometries. Then, exploiting the small-aspect ratio limit (ϵ≪1, i.e., the dimensions of the deformable element are large compared to the distance between the membrane and the upper plate) and strong in the fact that, for the membranes zeta=0, it is easy to obtain the following model which obviously does not take into account the fringing field:*

(26) $$\left\{\begin{array}{c} -\Delta u=\frac{\lambda }{{\left(1-u\right)}^{2}}\\ u=0,\;\;\;on\;\;\partial \Omega . \end{array}\right.$$

## 4. A Result Concerning the Existence of the Solution: An Approach Based on Upper and Lower Solutions

In this section, we ask ourselves whether the presence of f(r) in (20) substantially modifies the algebraic condition of existence already known in the literature. In this regard, the following result holds.

**Theorem** **1.** *Concerning the model* (20), *let us consider two continuously and twice differentiable functions, $u_{1}\left(r\right)$ and $u_{2}\left(r\right)$ as shown in Figure 1, both defined on [0,R], such that ∀r∈(0,R), $u_{1}\left(r\right)<u_{2}\left(r\right)$ in order that*

(27) $$\frac{du_{1}\left(r\right)}{dr^{2}}+r^{-1}\frac{du_{1}\left(r\right)}{dr}+4{\left(1-u_{1}\left(r\right)-d^{*}\right)}^{2}{\left(\theta \lambda f\left(r\right)\right)}^{-1}{\left(1+\delta |\frac{du_{1}\left(r\right)}{dr}{|}^{2}\right)}^{-1}>0,$$

*and*

(28) $$\frac{du_{2}\left(r\right)}{dr^{2}}+r^{-1}\frac{du_{2}\left(r\right)}{dr}+4{\left(1-u_{1}\left(r\right)-d^{*}\right)}^{2}{\left(\theta \lambda f\left(r\right)\right)}^{-1}{\left(1+\delta |\frac{du_{2}\left(r\right)}{dr}{|}^{2}\right)}^{-1}>0.$$

Moreover, if ∀r≠0, $4{\left(1-u_{1}\left(r\right)-d^{*}\right)}^{2}{\left(\theta \lambda f\left(r\right)\right)}^{-1}{\left(1+\delta |\frac{du(r)}{dr}{|}^{2}\right)}^{-1}$ is a continuous Lipschitzian function in $\{\left(r,u\right)\;:\;0<r<R\;\;and\;\;u_{1}\left(r\right)\leq u\left(r\right)\leq u_{2}\left(r\right)\}\times \left(-\infty ,+\infty \right)$ together with $f\left(r\right)\in L^{\infty }\left(\left[-R,R\right]\right)$, and

(29) $$\frac{du_{1}\left(0\right)}{dr}\geq \frac{du_{2}\left(0\right)}{dr},\;\;\;\;u_{1}\left(R\right)=u_{2}\left(R\right)=0,$$

together with the fact that the pull-in voltage $\lambda^{*}\;{∋}^{\prime }\;\;\forall \lambda \in \left(0,\lambda^{*}\right)$ exists. It follows that

(30) $$\theta \lambda f\left(r\right)>2{\left(d^{*}\right)}^{2}R^{2}{\left[k\epsilon_{0}V^{2}\left(1+\delta {\left({\left(d^{*}\right)}^{-2}R^{-2}k\epsilon_{0}V^{2}r\right)}^{2}\right)\right]}^{-1}$$

*This ensures that the problem (20) has at least one solution.*

**Proof** **of** **Theorem** **1.** See Appendix A. □

**Figure 2:**
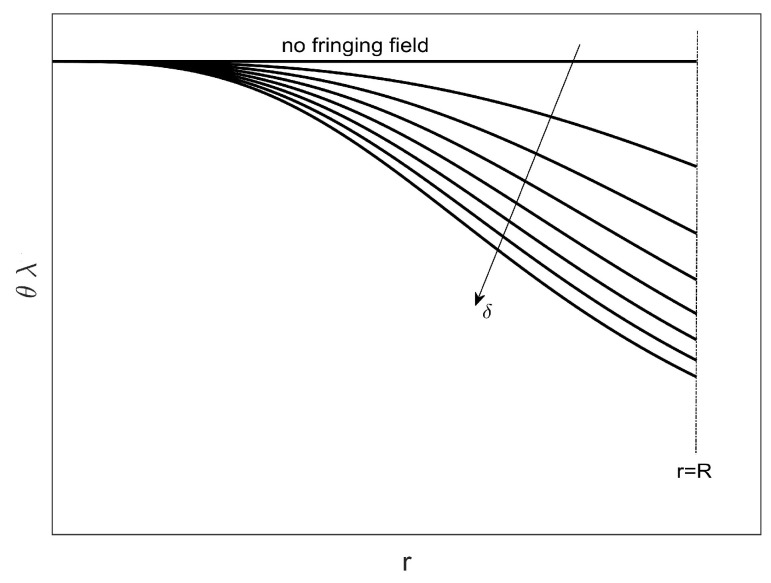
θλ versus *r*: under each curve, as δ increases, there is a forbidden area.

In Figure 2, one can see the trend of (30) as δ increases, which shows that the area under each curve (forbidden area) decreases as the effects due to the fringing field increase. There is a further decrease of the forbidden area when moving towards the edges of the device. However, unlike what was obtained in [51], the modeling of the dielectric properties of the membrane using f(r) modifies, by increasing the values of θλ, the extension of these areas by reducing the allowed areas on the r−θλ plane. It follows that, qualitatively, Figure 2 is a valid criterion for choosing the material constituting the membrane when the intended use of the micropump has been identified.

**Remark** **2.** *Unlike what was obtained in [51] where f(r)=1, here the boundedness of θλ is more accentuated; since the convergence of the numerical procedures used strongly depends on the minimum value assumed by θλ, the recovery of the membrane obtained here is much more realistic than that obtained in [51].*

## 5. A Result Concerning the Uniqueness of the Solution

In addition, regarding the uniqueness of the solution for (20), with respect to what was obtained in [51], a new algebraic condition is obtained. Formally, the following result holds.

**Theorem** **2.** *With the same hypotheses formulated in Theorem 1, the uniqueness of the solution for* (20) *is ensured if*

(31) $$\theta \lambda f\left(r\right)>\left(4R+R^{2}\right)\left(1+\delta H^{2}\right),$$

*where $H=\sup_{r\in (0,R]}|\frac{du(r)}{dr}|=|\frac{du(R)}{dr}|$.*

**Proof** **of** **Theorem** **2.** See Appendix B. □

As in [51], the dependence of (31) on *H* has an important practical implication. In fact, since the membrane deforms symmetrically with respect to the vertical axis passing through r=0, the greatest slope of the membrane profile is found at the edges, and this is confirmed by the numerical recovering performed below. However, unlike what was obtained in [51], here the uniqueness of the solution (governed by (31)) is ensured not only by the presence of θλ and by the geometry of the micropump but also by the presence of f(r), confirming that the dielectric properties strongly influence the deformation of the membrane. Therefore, the greater the f(r) value together with *R* and δ, the greater *V* must be in order to overcome the mechanical inertia of the membrane.

## 6. On the Existence and Uniqueness of the Solution

To achieve this condition, it is sufficient to solve the algebraic system constituted by both (30) and (31). It is easy to verify that

(32) $$2{\left(d^{*}\right)}^{2}R^{2}{\left[k\epsilon_{0}V^{2}\left(1+\delta {\left(k{\left(d^{*}\right)}^{-2}R^{-2}\epsilon_{0}V^{2}\right)}^{2}\right)\right]}^{-1}>4R\left(1+R\right)\left(1+\delta H^{2}\right).$$

because, on the contrary, one would obtain

(33) $$H>{\left(\delta^{-1}d^{*}R{\left[2\left(1+R\right)\epsilon_{0}V^{2}{\left(1+\delta k\epsilon_{0}{\left(d^{*}\right)}^{-2}R^{-2}V^{2}r\right)}^{2}\right]}^{-1}-1\right)}^{1/2}$$

which represents an implausible result. In fact, taking into account that H=146 (as already proven in [51]) when substituting in (33) a plausible value for each parameter, it would follow that $H>10^{12}$. Therefore, condition (30) ensures both the existence and uniqueness of the solution for (20) so that any numerical recovery of the membrane that does not satisfy it will give a ghost solution.

## 7. On the Stability of the Solutions

For this purpose, we will use the first Lyapunov’s criterion. Therefore, by exploiting two functions, $g_{1}\left(r\right)$ and $g_{2}\left(r\right)$, such that

(34) $$g_{1}\left(r\right)=g\left(r\right),\;\;\;\;g_{2}\left(r\right)=\frac{dg(r)}{dr},$$

model (20) can be rewritten as

(35) $$\left\{\begin{array}{c} \frac{dg_{1}\left(r\right)}{dr}=\bar{f}\left(g_{1}\left(r\right),g_{2}\left(r\right)\right)=g_{2}\left(r\right),\\ \frac{dg_{2}\left(r\right)}{dr}=\bar{g}\left(g_{1}\left(r\right),g_{2}\left(r\right)\right)=-r^{-1}g_{2}\left(r\right)-{\left(\theta \lambda f\left(r\right)\right)}^{-1}{\left(1+\delta |\frac{dg_{1}\left(r\right)}{dr}{|}^{2}\right)}^{-1}{\left(1-g_{1}\left(r\right)-d^{*}\right)}^{2},\\ u_{1}\left(R\right)=u_{2}\left(0\right)=0. \end{array}\right.$$

Therefore, according the fist Lyapunov’s criterion, by imposing $\frac{dg_{1}\left(r\right)}{dr}=\frac{dg_{2}\left(r\right)}{dr}=0$, we achieve the following critical point:

(36) $$\left(g_{1}^{0},g_{2}^{0}\right)=\left(1-d^{*},0\right).$$

Point (36) identifies the position of $u_{0}$ of the membrane next to the counter-electrode because $d^{*}$ is very small [51]. However, it is easy to see that

(37) $$p_{el}=0.5{\left(1-u\left(r\right)\right)}^{-2}\epsilon_{0}V^{2}\approx 0.5{\left(d^{*}\right)}^{-1}\epsilon_{0}V^{2},$$

so that, once *V* is selected, $p_{el}$ (and consequently *p*) is not affected by appreciable variation. Thus, by linearizing (35) around (36), since both $g_{1}\left(r\right)$ and $g_{2}\left(r\right)$ are analytical functions, and considering the following new variables (with a small enough ϵ)

(38) $$g_{1}\left(r\right)=g_{1}^{0}+\epsilon \xi \left(r\right);\;\;\;u_{2}\left(r\right)=g_{2}^{0}+\epsilon \eta \left(r\right),$$

the Taylor series made of both $\bar{f}\left(g_{1}\left(r\right),g_{2}\left(r\right)\right)$ and $\bar{g}\left(g_{1}\left(r\right),g_{2}\left(r\right)\right)$ (neglecting the terms of an order higher than the linear one) becomes

(39) $$\left\{\begin{array}{c} \frac{d\xi (r)}{dr}=\frac{\partial \bar{f}\left(g_{1}^{0},g_{2}^{0}\right)}{\partial g_{1}}\xi \left(r\right)+\frac{\partial \bar{f}\left(g_{1}^{0},g_{2}^{0}\right)}{\partial g_{2}}\eta \left(r\right)\\ \frac{d\eta (r)}{dr}=\frac{\partial \bar{g}\left(g_{1}^{0},g_{2}^{0}\right)}{\partial g_{1}}\xi \left(r\right)+\frac{\partial \bar{g}\left(g_{1}^{0},g_{2}^{0}\right)}{\partial g_{2}}\eta \left(r\right). \end{array}\right.$$

where $\tau =\sqrt{\xi^{2}+\eta^{2}}$. Furthermore, $\frac{\partial \bar{f}\left(g_{1}^{0},g_{2}^{0}\right)}{\partial g_{1}}=0$, $\frac{\partial \bar{f}\left(g_{1}^{0},g_{2}^{0}\right)}{\partial u_{2}}=1$, $\frac{\partial \bar{g}\left(g_{1}^{0},g_{2}^{0}\right)}{\partial g_{1}}=-r^{-1}$ and $\frac{\partial \bar{g}\left(g_{1}^{0},g_{2}^{0}\right)}{\partial u_{2}}=0$; thus (39) can be written as

(40) $$\left\{\begin{array}{c} \frac{d\xi (r)}{dr}=\eta \left(r\right)\\ \frac{d\eta (r)}{dr}=-r^{-1}\eta \left(r\right), \end{array}\right.$$

whose matrix has no eigenvalues with a positive real part; therefore, (36) represents a stable equilibrium position for (20), whatever f(r).

**Remark** **3.** It is worth noting that, unlike existence and uniqueness, the stability of solutions does not depend on f(r) but is strongly influenced by $d^{*}$. Then, the result obtained here can also be extended to [51], where f(r)=1.

## 8. How to Mathematically Model the Dielectrical Properties of the Membrane

As highlighted in [56,57,58,59,60,61], pull-in instability could occur in this kind of device, regardless of f(r). Therefore, here, we question where there are formulations for f(r) that affect u(r), which is usually symmetrical and concave with u(r)<1. Experimentally, the area of the membrane around $u_{0}$ could present instability because, according to (19), |E| is very strong ($1-u\left(r\right)-d^{*}\ll 1$) while on the edge it is very weak (u(r)=u(R)=0). Thus, we need to formulate f(r) to reduce |E| at r=0 by allowing it to be stronger near the edges. A good formulation for f(r), as a large experimental application highlight, seems to be [58,59,60,61]

(41) $$f\left(r\right)={\left|r\right|}^{\alpha },\;\;\;\alpha \geq 0,$$

so that

(42) $$\left|E\right|\propto {\left(1-u\left(r\right)\right)}^{-2}\lambda {\left|r\right|}^{\alpha },$$

which represents the electrostatic load function for the micropump. However, we question whether specific formulations of f(r) can affect the numerical solutions (and any multiplicity) without neglecting the pull-in voltage and the stable operating range of the micropump.

**Remark** **4.** *From* (32), *it is easy to achieve the following limitation for H:*

(43) $$H<\sqrt{\delta^{-1}\left(2{d^{*}}^{2}\right)R^{2}{\left(4R\left(1+R\right)\right)}^{-1}(k\epsilon_{0}V^{2}(1+\delta {\left(k\epsilon_{0}V^{2}{d^{*}}^{-1}R^{-2}\right)}^{-1}-1}$$

*from which the following inequality*

(44) $$\delta \leq {d^{*}}^{2}R^{2}{\left(2R\left(1+R\right)\right)}^{-1}(k\epsilon_{0}V^{2}{\left(1+\delta k\epsilon_{0}V^{2}{\left(d^{*}R^{2}\right)}^{-1}\right)}^{-1},$$

*which, solved, gives us*

(45) 0≤δ≤1.91,

*which, unlike what was obtained in [51] (where 0≤δ≤2), shows how the definition of the dielectric profile of the membrane lowers the maximum value to be assigned to the parameter and weighs the effects due to the fringing field.*

**Remark** **5.** *Regarding the fringing field, $E_{\mathrm{fringing}\;\mathrm{field}}$, we can write*

(46) $$\begin{array}{c} 0.25\delta \theta \lambda f\left(r\right){\left(1-u\left(r\right)-d^{*}\right)}^{-2}|\frac{du(r)}{dr}{|}^{2}{\left(\frac{d^{2}u\left(r\right)}{dr^{2}}+r^{-1}\frac{du(r)}{dr}\right)}^{2}=\\ =\gamma |E_{\mathrm{fringing}\;\mathrm{field}}{|}^{2},\;\;\;\gamma \in R^{+} \end{array}$$

*From this, considering $f_{el}$ and exploiting (4), the total electrostatic force is computable using*

(47) $$\begin{array}{c} F_{el_{T}}=f_{el_{\mathrm{fringing}\;\mathrm{field}}}+f_{el}=\\ =0.125{\left(1-u\left(r\right)-d^{*}\right)}^{-2}\epsilon_{0}\pi R^{2}\delta \theta \lambda f\left(r\right)|\frac{du(r)}{dr}{|}^{2}{\left(\frac{d^{2}u\left(r\right)}{dr^{2}}+r^{-1}\frac{du(r)}{dr}\right)}^{2}+\\ +0.5{\left(d-u\left(r\right)-d^{*}\right)}^{-2}\epsilon_{0}\pi R^{2}V^{2}. \end{array}$$

*Therefore, by using the conditions (4) and $|\frac{du(r)}{dr}|<146$ [49] and substituting the usual value for each physical parameter, we can obtain the following inequality that depends on both V and T*

(48) $$F_{el_{T}}<\left(32\cdot 10^{39}+12\cdot 10^{-12}\delta \right)T^{-1}V^{2},$$

*This shows that the greater T ($T=\sup_{r}\left\{T\left(r\right)\right\}$, bounded function) is, the smaller the effect due to the fringing field is (as experimentally confirmed [58,59,61]). Moreover, we observe that (48) is similar to the link obtained in [52] where a plate represents the deformable element. This underlines the fact that the approach used allows for obtaining a general limitation for the total electrostatic force inside the device which can be calculated through a general formulation regardless of whether the deformable element is a plate or a membrane (obviously, depending on the cases, the values to be assigned to each parameter change). Finally, it should be noted that the effects due to the fringing field are negligible as evidenced by the coefficient of δ in (48). Thus, both the intended use of the device and the mechanical properties of the deformable plate influence the behavior of the micropump.*

## 9. Numerical Recovery of the Membrane Profile

Here, we exploit numerical techniques considered the “gold standard” for this type of problem with a singularity of 1/r [51]. Particularly, the Keller–Box scheme was implemented in MatLab R2022 together with shooting techniques and the III/IV Lobatto IIIa formulas, which were respectively implemented by exploiting the subroutines ode23, ode45, bpv4c, and bpv5c (mathematical details can be easily found in [51,52]). The Keller–Box procedure, although computationally expensive, offers a better performance. This computational load could create problems in special real-time applications. However, there are very few applications where near-instantaneous membrane recovery is required.

We highlight that, by retracing the procedure used in [51], it is easy to achieve

(49) $$\begin{array}{c} \theta \lambda \geq {\left|r\right|}^{-\alpha }{\left(d^{*}\right)}^{6}R^{6}V^{-2}{\left(1+4\delta d^{2}{\left(1-u\left(r\right)\right)}^{4}\right)}^{-1}>\\ >V^{-2}A{\left(d^{*}\right)}^{6}R^{6}{\left(1+4\delta d^{2}{\left(1d-u\left(r\right)\right)}^{4}\right)}^{-1},\;\;\;A\in R^{+}, \end{array}$$

from which, for each numerical approach *j*, even in the presence of a fringing field and in convergence conditions, the corresponding profile of the membrane, $u_{j,\delta }\left(r\right)$ satisfying (49) does not represent a ghost solution. Therefore, the range of values that ensure convergence without ghost solutions is ∀j and ∀δ, $\left[{\left({\left(\theta \lambda \right)}_{conv-\;no\;ghost\;solutions}\right)}_{j,\delta },+\infty \right)$, where

(50) $$\begin{array}{c} {\left({\left(\theta \lambda \right)}_{conv-\;no\;ghost\;solutions}\right)}_{j,\delta }=\\ =AV^{-2}{\left(d^{*}\right)}^{6}R^{6}{\left(1+4\delta d^{2}{\left(d-\max_{r}\left\{\max_{j}\left\{u_{j,\delta }\left(r\right)\right\}\right\}\right)}^{4}\right)}^{-1}. \end{array}$$

Obviously, if $\theta \lambda >\inf{\left(\theta \lambda \right)}_{\mathrm{conv}}$, all of the numerical procedures will converge, but one must pay attention to possible ghost solutions. However, unlike what was obtained in [51], given the presence of f(r), there is an increase in the values of θλ which determine the convergence intervals (even as the of δ). Table 1 highlights the range of possible values for θλ when α=0.2, which ensures the convergence of all numerical procedures without ghost solutions (i.e., the numerical solutions obtained satisfy the condition (30)). Indeed, all maximum values of *u* obtained numerically (denoted by $u_{i}$) satisfy (30).

**Figure 3:**
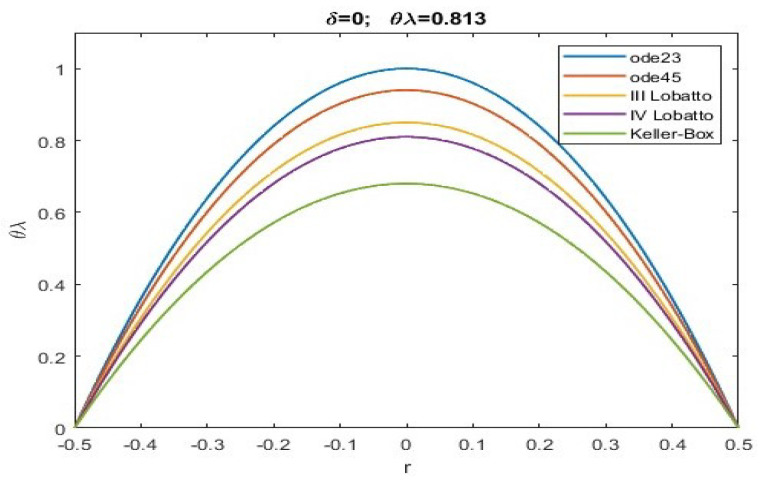
Recovering of u(r) for δ=0 and θλ=0.813.

**Figure 4:**
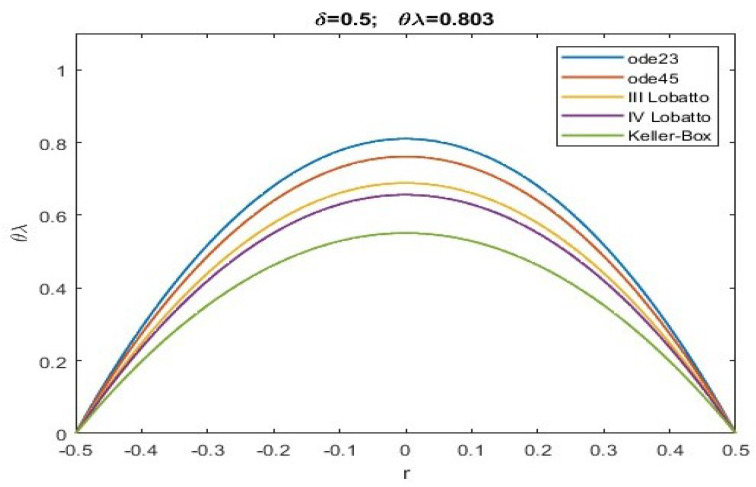
Recovering of u(r) for δ=0.5 and θλ=0.803.

**Figure 5:**
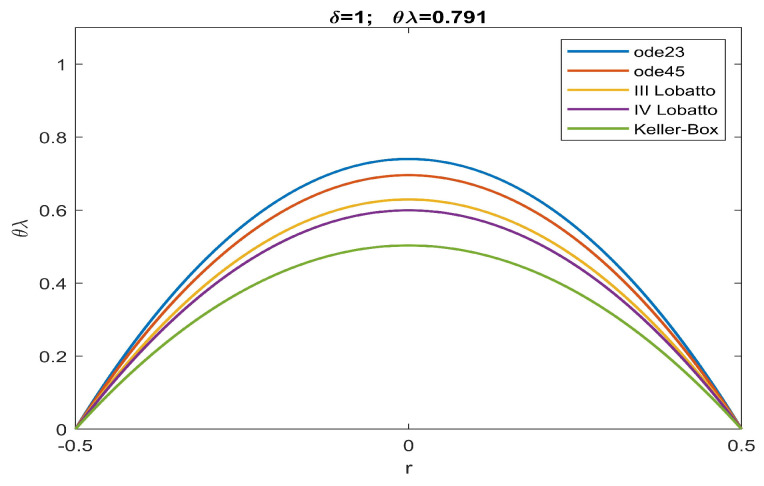
Recovering of u(r) for δ=1 and θλ=0.791.

**Figure 6:**
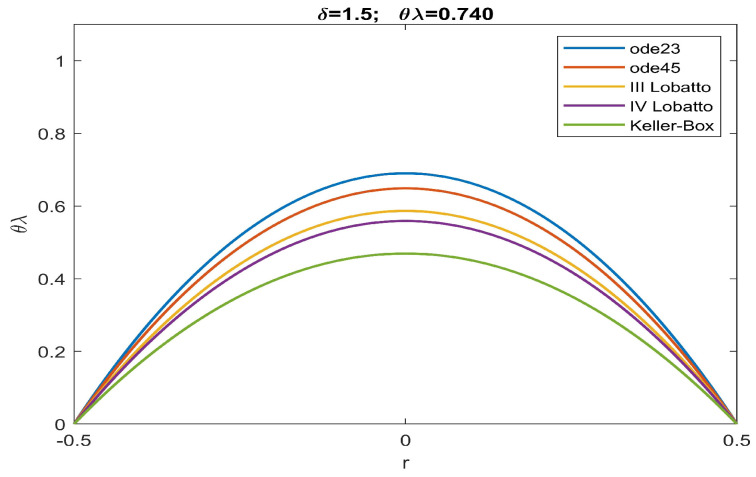
Recovering of u(r) for δ=1.5 and θλ=0.740.

**Figure 7:**
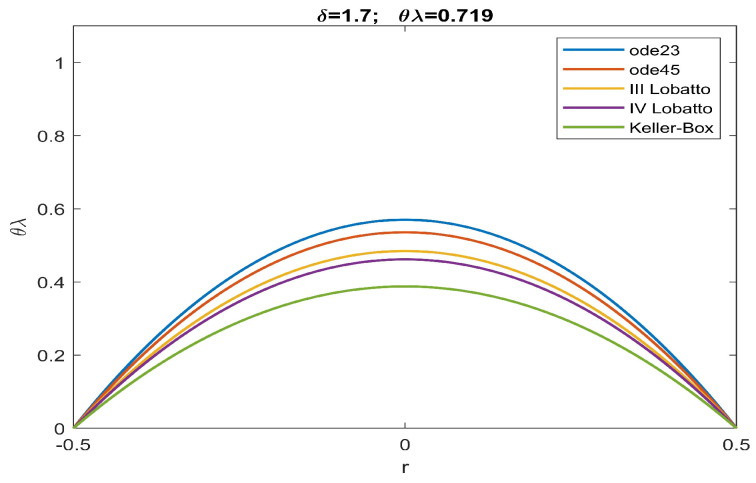
Recovering of u(r) for δ=1.7 and θλ=0.719.

From the analysis of the numerical data obtained, agreement with the qualitative results highlighted in Figure 2 is evident. According to this, there is an increase in the values of θλ compared to what was obtained in [51]. More specifically, the more intense the effects caused by the fringing field, the lower are the minimum values of the intervals of θλ capable of guaranteeing the convergence of numerical procedures both in the presence and absence of ghost solutions. Our study highlighted how the numerical procedure based on a Keller–Box scheme provides a minimum value of θλ which guarantees that all numerical procedures converge without the profiles of the recovered membrane representing ghost solutions (see Table 1). Finally, we underline the fact that both Keller–Box and Lobatto’s formulas were set with a number of nodes equal to 45 (below which Keller–Box did not converge, while both Lobatto’s formulas provided inaccurate results) to allow the results to be compared with those set automatically by ode23 and ode45. In Figure 3, Figure 4, Figure 5, Figure 6 and Figure 7, the profiles of the membrane obtained numerically are displayed, increasing δ in compliance with the range of possible values for θλ and ensuring the convergence of all the numerical procedures. From these figures, it is clear that, as δ increases, $u_{0}$ significantly decreases, as required in (20). Furthermore, the approach used here, reducing θλ (i.e., reducing externally applied *V*) establishes regimes of small membrane displacements allowing regular and symmetrical recovery of the membrane with limited deformations, avoiding tearing of the membrane especially at the edges (in [51], the recovery of the membrane showed high entity trapezoidal deformations) favouring the administration of small amount of drugs, as required in specific therapies.

## 10. External Voltage Needed to Overcome the Mechanical Inertia of the Membrane

As anticipated above, from (30), with r<R, by exploiting (4), it is easy to achieve

(51) $$\min\left\{V\right\}={\left(d^{3}d^{*4}T{\left|r\right|}^{\alpha }{\left(\theta \epsilon_{0}k\left(d^{*2}+\delta k^{2}\right)\right)}^{-1}\right)}^{1/4}$$

which represents the externally applied *V* that is necessary to overcome the mechanical inertia of the membrane ((51) is analogous to the one obtained in [51] but with the presence of f(r)). From (51), it can be seen that the presence of fringing field, by curving the lines of force of E towards the outside of the micropump, favors the deformation of the membrane, so the device is also recommended for the administration of low-dose drugs [5,6].

## 11. On the Selection of the Material Constituting the Membrane and the Intended Use of the Micropump

**Figure 8:**
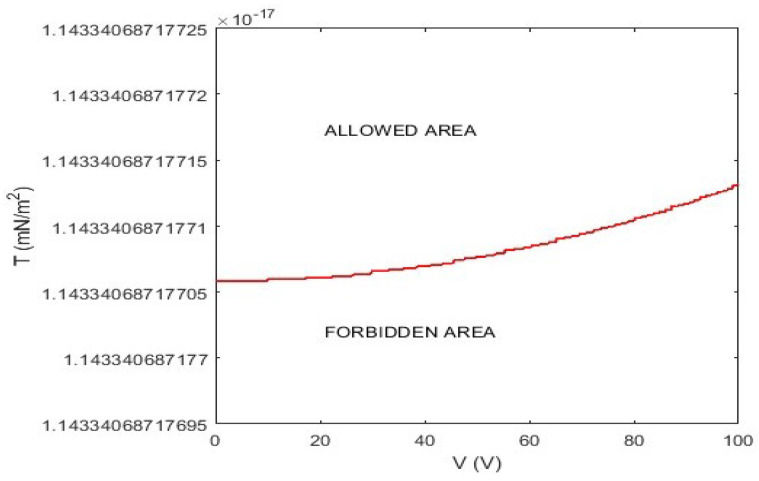
Graphic representation of (52) to highlight the (V,T) pairs that allow numerical recovery.

From (51) it can be deduced that the choice of the material constituting the membrane (i.e., *T*) strongly conditions the minimum value of volts to be attributed to the external source in order to overcome the inertia of the membrane itself. We ask ourselves what condition *T* and *V* must satisfy to ensure the correct functioning of the device. Therefore, by retracing the same approach used in [51], it is possible to achieve

(52) $$\begin{array}{c} 0.25\epsilon_{0}V^{4}\left(1+\delta H^{2}\right){\left(|r\right|}^{\alpha }T^{2}{\sup\{|E|}^{2}{\})}^{-1}>\\ >\min_{j}\left\{{\left({\left(\theta \lambda \right)}_{conv-\;no\;ghost\;solutions}\right)}_{j,\delta }\right\}, \end{array}$$

from which the link between *T* (which represents the material constituting the membrane) and *V* (which represents the intended use of the micropump) depends on both the electromechanical and dielectric properties of the membrane, θ and λ, which influence both the concavity of u(r) (the greater the *T*, the lower the concavity) and the convergence of the numerical procedures. This confirms what has already been highlighted in [50], where a similar micropump had been studied in a one-dimensional regime. The results obtained here, with the necessary variations due to f(r), are completely superimposable since the model has evident radial symmetry. Moreover, from (52), according to [50,51], it can be seen that the highly stressed membranes have a greatly reduced risk of ghost solutions as they allocate the device to applications with low electrical potential values. Particularly, by setting the possible value for each parameter, a graphical representation of (52) is shown in Figure 8, in which both *T* and *V* are no longer dimensionless and both allowed and forbidden areas are depicted. Furthermore, from Figure 8, it can be seen that the modeled micropump allows a wide range of uses, since the allowable *T* of the membrane has an extremely low minimum allowed value. The trend displayed in Figure 8 is similar to that obtained in [52] where the link between *T* and *V* assumes the same trend but with more high numerical values because it was considered a MEMS device whose deformable element was a plate.

## 12. Some Considerations for Both the Pull-In Voltage and the Electrostatic Pressure

**Figure 9:**
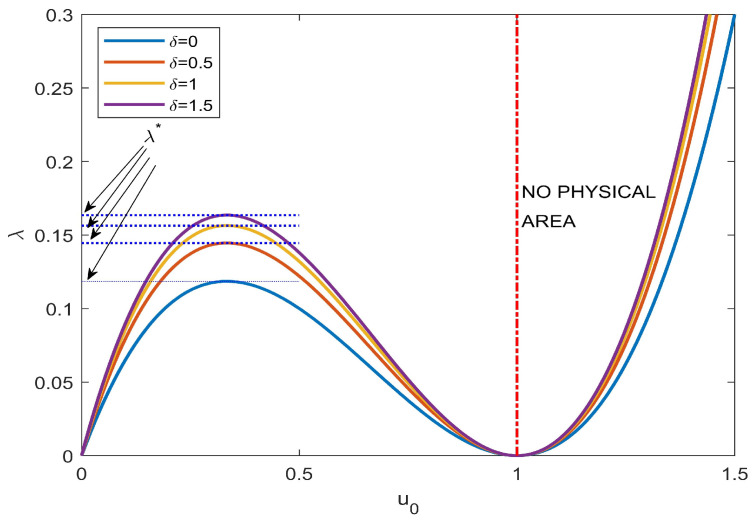
Dimensionless $\lambda^{*}$ as a function of a dimensionless $u_{0}$.

High values of *V* (beyond the “pull-in” value) could create instability due to high electrostatic force values that exceed the elastic ones [57,58]. Specifically, if $\lambda >\lambda^{*}$, (20) does not provide solutions (for details, see [55,57,58,62,63]). Starting from λ=0, we traced the trends of $u_{0}$ to highlight the values of $\lambda^{*}$ as a function of δ. The values above $\lambda^{*}$ show that (20) has no solution. Figure 9, starting from the absence of a fringing field, highlights the trend of the bifurcation diagram, which has characteristics similar to the experimental evidence [53,55].

**Figure 10:**
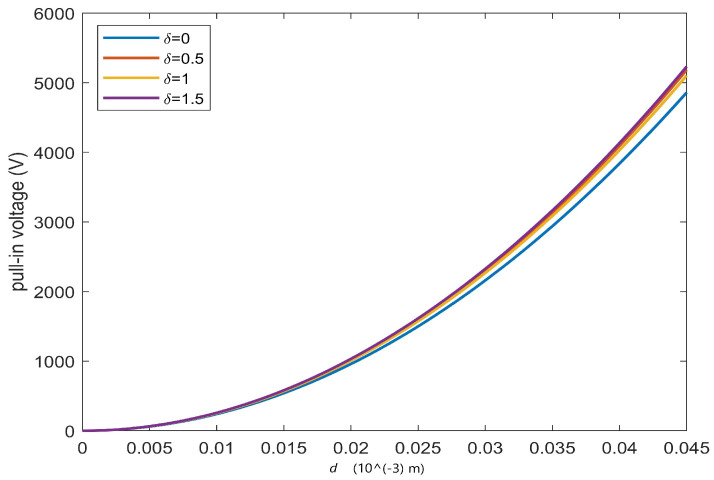
Pull-in voltage as function of the distance *d*.

To confirm this, Figure 10 displays the trend of the pull-in voltage, $V_{pull-in}$, as a function of the space between the two electrodes. It appears qualitatively superimposable to what is reported in [53]. It should be noted that the increase of *d* produces a deviation of the pull-in voltage, as δ varies. Then, as the effects due to the fringing field increase, the pull-in voltage values rise, removing the risk of bifurcations. Moreover, from (4), it is easy to achieve

(53) $$V_{pull-in}={\left(2Td^{3}\lambda^{*}{\left(\epsilon_{0}{\left(2R\right)}^{2}\right)}^{-1}\right)}^{-1/2}$$

which is very similar to the analogous formulation presented in [53].

Furthermore, from (47) together with (4), it is very easy to achieve $p_{el}$ due to the fringing field effects:

(54) $${\left(p_{el}\right)}_{fringing}=\epsilon_{0}\delta V^{2}|\frac{du(r)}{dr}{|}^{2}{\left(T{\left(1-u\left(r\right)-d^{*}\right)}^{2}\right)}^{-1}{\left(2R\right)}^{2}d^{-3},$$

**Figure 11:**
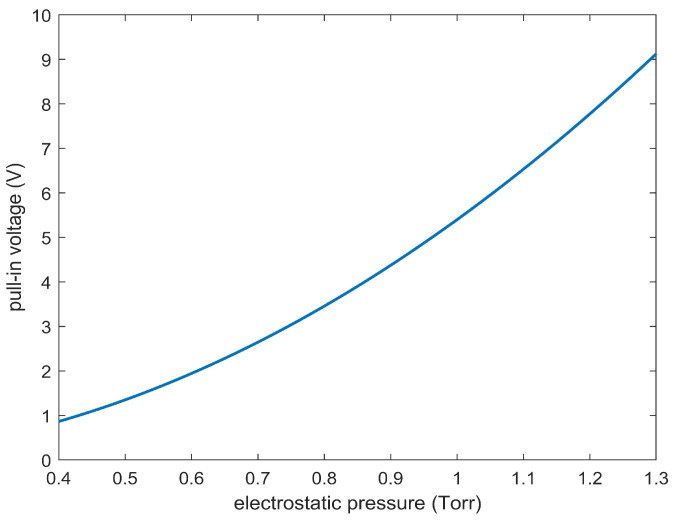
$V_{pull-in}$ versus ${\left(p_{el}\right)}_{fringing}$.

With (53), this gives us the link between ${\left(p_{el}\right)}_{fringing}$ and $V_{pull-in}$, as displayed in Figure 11. It is worth noting that (54) is completely analogous to the one obtained in [52,64] where the deformable element was a metallic plate.

## 13. Conclusions and Perspectives

Biomedical micropumps for intravenous drug delivery represent an important facility when treatment is reserved for uncooperative patients. Recently, devices have been developed with physics that fall into various fields such as electrostatic micropumps that are of significant importance, since they are easy to make and do not require particular maintenance operations. Physical-mathematical modeling currently exhibits models that realistically describe the behavior of the device in various operating phases. However, given their complexity, they are not suitable for any real-time applications. Circular electrostatic membrane micropumps are no exception, and their analytical models present singularities. In this work, a dimensionless 2D model with second-order differential circular symmetry was presented and studied to model an electrostatic membrane micropump for biomedical purposes, and the effects due to the fringing field (according to Pelesko–Driscoll theory which allows the addition of just one a term via software/hardware) were presented. The modeling of the device, which is voltage controlled by a suitable capacitive electric circuit, expresses |E| in terms of the mean curvature of the membrane to allow the use of important results for the study of the existence, uniqueness, and stability of the solution. Therefore, results concerning the existence, uniqueness, and stability of the solution were proved, and the dielectric properties of the material constituting the membrane (here modeled to simulate the capacitive effects during the deformation of the membrane) were exploited. These are strongly linked to the intended use of the device (links to be improved using more sophisticated formulations of curvatures). By using appropriate numerical techniques, the recovery of the membrane was carried out for different electrostatic load conditions, highlighting limitations for some electrical and mechanical quantities, including the electric voltage and the electrostatic force, and highlighting good overlap with experimental evidence in the literature. This allows the recovery of membrane profiles that are advantageous in terms of the safety of the micropump (absence of contact of the membrane with the counter-electrode).

Numerical recovery in our work is a means of recognizing any ghost solutions and understand the link between the properties of the constituent material the membrane and any intended use of the device in the various operating conditions. Thus, it is necessary in the near future to proceed with a FEM analysis of the analytical model under study in order to obtain a software tool that can be easily translated into hardware for any industrial prototypes. In this work, we have limited ourselves to using the numerical procedures since, currently, they are considered a “gold standard” for this type of analytical problems. Furthermore, a criterion for choosing the intended use of the micropump was proposed on the basis of the mechanical tension of the membrane at rest being an important limitation associated with using both *V* and *T* to reconstruct u(r) (model solution) while providing complete safety. The results obtained are comparable with experimental evidence in the literature, especially for cases where small displacements of the membrane are required. The work represents a step of a research project that has the main objective of investigating physical-mathematical models of both membrane and deformable plate microdevices. The cornerstone of this project is the physical-mathematical idea according to which the amplitude of the electric field inside the electrostatic device is locally proportional to the average curvature of the deformable element at the point considered. Specifically, the submitted paper developed the step concerning the 2D modeling of a membrane device in the presence of a modeled fringing field, through one of the most accredited theories developed by Pelesko and Driscoll, with relative numerical recovery of the deformable element and analysis of possible intended uses of the device starting from some properties of the membrane itself. At present, we do not have sufficient elements to define with certainty any practical and specific applications for this type of device. However, we are certain that the proposed device, at least in theory, can be used in biomedical applications in which the external voltage applied is reduced. In the near future, once the dependence of δ on *V* has been defined and formalized, it will be desirable to develop more complete models (including the dynamic aspect of the problem and the dependence on the electrical conductivity and on the temperature) so that the adherence with the experimental evidence is more marked.

## Figures and Tables

**Table 1:** Range of θλ ensuring convergence without ghost solutions.

δ	ode23	ode45	Keller	bpv4c	bpv5c	$\max\{u_{i}\}$	(30)
			Box				
0	0.702	0.694	0.813	0.745	0.732	0.97	verified
0.5	0.659	0.679	0.803	0.701	0.711	0.84	verified
1	0.623	0.648	0.791	0.691	0.703	0.77	verified
1.5	0.591	0.622	0.740	0.684	0.699	0.72	verified
1.7	0.543	0.619	0.719	0.655	0.681	0.63	verified

## Data Availability

Not applicable.

## Appendix A

**Proof** **of** **Theorem** **1.** We preliminarily observe that $f\left(r\right)\in L^{\infty }\left(\left[-R,R\right]\right)$ means that ${\left||f|\right|}_{\infty }=\inf\left\{S\geq 0\;\;:f\left(r\right)\leq S\;\;\;a.e.\right\}$ so that f(r) is a positive bounded function. Before proving Theorem 1, we note that (20) is a particular case of the following model:

(A1) $$\left\{\begin{array}{c} \frac{d^{2}u\left(r\right)}{dr^{2}}+F\left(r,u\left(r\right),\frac{du(r)}{dr},f\left(r\right)\right)=0\\ u\left(b\right)=B,\;\;\;\;\frac{du(a)}{dr}=m, \end{array}\right.$$

where m,B∈R and

(A2) $$\begin{array}{c} F\left(r,u\left(r\right),\frac{du(r)}{dr},f\left(r\right)\right)=\\ =r^{-1}\frac{du(r)}{dr}+4{\left(1-u\left(r\right)-d^{*}\right)}^{2}{\left[\theta \lambda f\left(r\right)\left(1+\delta |\frac{du(r)}{dr}{|}^{2}\right)\right]}^{-1}, \end{array}$$

belongs to $C^{0}\left(\left(a,b\right]\times R\times R\right)$. (A1) allows us to exploit the Lemma A1 proved in [49]. □

**Lemma** **A1.** *About* (20), *we consider two twice continuously differentiable functions, $u_{1}\left(r\right)$ and $u_{2}\left(r\right)$, such that, ∀r∈(a,b), $u_{1}\left(r\right)<u_{2}\left(r\right)$ with (a,b). Let us also consider*

(A3) $$\frac{d^{2}u_{1}\left(r\right)}{dr^{2}}+F\left(r,u_{1}\left(r\right),\frac{du_{1}\left(r\right)}{dr}\right)>0,\;\;\;\frac{d^{2}u_{2}\left(r\right)}{dr^{2}}+F\left(r,u_{2}\left(r\right),\frac{du_{2}\left(r\right)}{dr}\right)<0,$$

*Furthermore, let $F\left(r,y\left(r\right),\frac{dy(r)}{dr}\right)$ be a Lipschitzian continuous function, that is:*

(A4) $$\begin{array}{c} K_{1}\left(r\right)\left(u\left(r\right)-v\left(r\right)\right)+L_{2}\left(r\right)\left(\frac{du(r)}{dr}-\frac{dv(r)}{dr}\right)\leq \\ \leq F\left(r,u\left(r\right),\frac{du(r)}{dr}\right)-F\left(r,v\left(r\right),\frac{dv(r)}{dr}\right)\leq \\ \leq K_{2}\left(r\right)\left(u\left(r\right)-v\left(r\right)\right)+L_{1}\left(r\right)\left(\frac{du(r)}{dr}-\frac{dv(r)}{dr}\right) \end{array}$$

*in $\{\left(r,u\right)\;\;:\;\;a<r<b\;\;\mathrm{and}\;\;u_{1}\left(r\right)\leq u\left(r\right)\leq u_{2}\left(r\right)\}\times \left(-\infty ,+\infty \right)$ where $K_{1}\left(r\right)$, $K_{2}\left(r\right)$, $L_{1}\left(r\right)$ and $L_{2}\left(r\right)$ are continuous functions in (a,b]. Moreover, if $\frac{du_{1}\left(a\right)}{dr}\geq \frac{du_{2}\left(a\right)}{dr}$, with $u_{1}\left(b\right)=u_{2}\left(b\right)=B$,* (A1) *has at least one solution such that $u_{1}\left(r\right)\leq u\left(r\right)\leq u_{2}\left(r\right)$ in [a,b].*

**Proof** **of** **Theorem** **4.** Exploit Lemma A1, we can assume that, ∀r∈[0,R] [50],

(A5) $$u_{1}\left(r\right)=0,$$

and

(A6) $$u_{2}\left(r\right)=\bar{u}\left(r\right)=0.5k\epsilon_{0}{\left(d^{*}\right)}^{-2}V^{2}\left(1-{\left(rR^{-1}\right)}^{2}\right).$$

as displayed in Figure 1 in which a possible recovering of the membrane is also possible, which is shown in red. Obviously, both $u_{1}\left(r\right)$ and $u_{2}\left(r\right)$ are twice continuously differentiable functions such that $u_{1}\left(r\right)<u_{2}\left(r\right)$. Observing that

(A7) $$\frac{du_{1}\left(r\right)}{dr}=\frac{d^{2}u_{1}\left(r\right)}{dr^{2}}=0,$$

therefore,

(A8) $$\begin{array}{c} \frac{d^{2}u_{1}\left(r\right)}{dr^{2}}+r^{-1}\frac{du_{1}\left(r\right)}{dr}+4{\left(1+\delta |\frac{du_{1}\left(r\right)}{dr}{|}^{2}\right)}^{-1}{\left(1-u_{1}\left(r\right)-d^{*}\right)}^{2}{\left(\theta \lambda f\left(r\right)\right)}^{-1}=\\ ={\left(\theta \lambda f\left(r\right)\right)}^{-1}{\left(1-d^{*}\right)}^{2}>0. \end{array}$$

Thus, (27) is verified. Moreover, from (A6), we can write

(A9) $$\frac{du_{2}\left(r\right)}{dr}=-k{\left(d^{*}\right)}^{-2}R^{-2}\epsilon_{0}V^{2}r,\;\;\;\;\frac{d^{2}u_{2}\left(r\right)}{dr^{2}}=-k{\left(d^{*}\right)}^{-2}R^{-2}\epsilon_{0}V^{2}$$

so that

(A10) $$\begin{array}{c} \frac{d^{2}u_{2}\left(r\right)}{dr^{2}}+r^{-1}\frac{du_{2}\left(r\right)}{dr}+4{\left(\theta \lambda f\left(r\right)\right)}^{-1}{\left(1-u_{2}\left(r\right)-d^{*}\right)}^{2}{\left(1+\delta |\frac{du(r)}{dr}{|}^{2}\right)}^{-1}=\\ =-2k\epsilon_{0}V^{2}d{*}^{-2}R^{-2}+\\ +4{\left(\theta \lambda f\left(r\right)\right)}^{-1}{\left(1-0.5k\epsilon_{0}V^{2}{\left(d^{*}\right)}^{-2}\left(1-{\left(rR^{-1}\right)}^{2}\right)-d^{*}\right)}^{2}\cdot \\ \cdot {\left(1+\delta (k\epsilon_{0}V^{2}r{\left(d^{*}\right)}^{-2}R^{-2}\right)}^{2}{)}^{-1}<0. \end{array}$$

with f(r) being a positive bounded function, is also a bounded function; then, ${\left(f\left(r\right)\right)}^{-1}<B$, with $B\in R^{+}$, and both θ and λ positive values, (A10) becomes

(A11) $$\begin{array}{c} 4[\theta \lambda f\left(r\right){\left(1+\delta \left(k\epsilon_{0}V^{2}r{\left({\left(d^{*}\right)}^{2}R^{2}\right)}^{-1}{)}^{2}\right)\right]}^{-1}\cdot \\ \cdot {\left(1-k\epsilon_{0}V^{2}{\left(2{\left(d^{*}\right)}^{2}\right)}^{-1}\left(1-{\left(rR^{-1}\right)}^{2}\right)-d^{*}\right)}^{2}<2{\left({\left(d^{*}\right)}^{2}R^{2}\right)}^{-1}k\epsilon_{0}BV^{2}. \end{array}$$

To verify (28), it is sufficient that

(A12) $$0\leq (1-k\epsilon_{0}V^{2}{\left(2{\left(d^{*}\right)}^{2}\right)}^{-1}\left(1-{\left(rR^{-1}\right)}^{2}\right)-d^{*}{)}^{2}<1,$$

so, by (A11),

(A13) $$\begin{array}{c} {\left(\theta \lambda f\left(r\right)\right)}^{-1}{\left(1+\delta {\left({\left(d^{*}\right)}^{-2}R^{-2}k\epsilon_{0}V^{2}r\right)}^{2}\right)}^{-1}<0.5{\left(d^{*}\right)}^{-2}R^{-2}k\epsilon_{0}BV^{2}. \end{array}$$

To verify (A4), we write:

(A14) $$\begin{array}{c} F\left(r,u\left(r\right)-v\left(r\right),\frac{du(r)}{dr}-\frac{dv(r)}{dr}\right)=\\ =r^{-1}\frac{du(r)}{dr}+4{\left[\theta \lambda f\left(r\right)\left(1+\delta |\frac{du(r)}{dr}{|}^{2}\right)\right]}^{-1}{\left(1-u\left(r\right)-d^{*}\right)}^{2}-\\ -r^{-1}\frac{dv(r)}{dr}-4{\left[\theta \lambda f\left(r\right)\left(1+\delta |\frac{dv(r)}{dr}{|}^{2}\right)\right]}^{-1}{\left(1-v\left(r\right)-d^{*}\right)}^{2}=\\ =r^{-1}\left(\frac{du(r)}{dr}-\frac{dv(r)}{dr}\right)+{\left(\theta \lambda f\left(r\right)\right)}^{-1}(4{\left[\theta \lambda f\left(r\right)\left(1+\delta |\frac{du(r)}{dr}{|}^{2}\right)\right]}^{-1}\cdot \\ \cdot {\left(1-u\left(r\right)-d^{*}\right)}^{2}-4{\left[\theta \lambda f\left(r\right)\left(1+\delta |\frac{dv(r)}{dr}{|}^{2}\right)\right]}^{-1}{\left(1-v\left(r\right)-d^{*}\right)}^{2})=\\ =r^{-1}\left(\frac{du(r)}{dr}-\frac{dv(r)}{dr}\right)+4{\left(\theta \lambda f\left(r\right)\right)}^{-1}({\left(1-u\left(r\right)-d^{*}\right)}^{2}\cdot \\ \cdot \left(1+\delta |\frac{du(r)}{dr}{|}^{2}-\delta |\frac{du(r)}{dr}{|}^{2}\right){\left(1+\delta |\frac{du(r)}{dr}{|}^{2}\right)}^{-1}-\\ -{\left(1-v\left(r\right)-d^{*}\right)}^{2}\left(1+\delta |\frac{dv(r)}{dr}{|}^{2}-\delta |\frac{dv(r)}{dr}{|}^{2}\right){\left(1+\delta |\frac{dv(r)}{dr}{|}^{2}\right)}^{-1})=\\ =r^{-1}\left(\frac{du(r)}{dr}-\frac{dv(r)}{dr}\right)+4{\left(\theta \lambda f\left(r\right)\right)}^{-1}({\left(1-u\left(r\right)-d^{*}\right)}^{2}\cdot \\ \cdot \left(1-{\left(1+\delta |\frac{du(r)}{dr}{|}^{2}\right)}^{-1}\delta |\frac{du(r)}{dr}{|}^{2}\right)-\\ -{\left(1-v\left(r\right)-d^{*}\right)}^{2}\left(1-\delta |\frac{dv(r)}{dr}{|}^{2}{\left(1+\delta |\frac{du(r)}{dr}{|}^{2}\right)}^{-1}\right))=\\ =r^{-1}\left(\frac{du(r)}{dr}-\frac{dv(r)}{dr}\right)+4{\left(\theta \lambda f\left(r\right)\right)}^{-1}({\left(1-u\left(r\right)-d^{*}\right)}^{2}-{\left(1-v\left(r\right)-d^{*}\right)}^{2}-\\ -{\left(1-u\left(r\right)-d^{*}\right)}^{2}\delta |\frac{du(r)}{dr}{|}^{2}{\left(1+\delta |\frac{du(r)}{dr}{|}^{2}\right)}^{-1}+\\ +{\left(1-v\left(r\right)-d^{*}\right)}^{2}\delta |\frac{dv(r)}{dr}{|}^{2}{\left(1+\delta |\frac{dv(r)}{dr}{|}^{2}\right)}^{-1}). \end{array}$$

However, being ${\left(1-u\left(r\right)-d^{*}\right)}^{2}\delta |\frac{du(r)}{dr}{|}^{2}>0$ and $1+\delta |\frac{du(r)}{dr}{|}^{2}$,

(A15) $${\left(1-u\left(r\right)-d^{*}\right)}^{2}\delta |\frac{du(r)}{dr}{|}^{2}{\left(1+\delta |\frac{du(r)}{dr}{|}^{2}\right)}^{-1}>0,$$

so that (A14) becomes:

(A16) $$\begin{array}{c} F(r,u\left(r\right)-v\left(r\right),\frac{du(r)}{dr}-\frac{dv(r)}{dr})<\\ <r^{-1}\left(\frac{du(r)}{dr}-\frac{dv(r)}{dr}\right)+4{\left(\theta \lambda f\left(r\right)\right)}^{-1}({\left(1-u\left(r\right)-d^{*}\right)}^{2}-{\left(1-v\left(r\right)-d^{*}\right)}^{2}+\\ +{\left(1-v\left(r\right)-d^{*}\right)}^{2}\delta |\frac{dv(r)}{dr}{|}^{2}{\left(1+\delta |\frac{dv(r)}{dr}{|}^{2}\right)}^{-1}). \end{array}$$

Furthermore, $\delta |\frac{dv(r)}{dr}{|}^{2}<1+\delta |\frac{dv(r)}{dr}{|}^{2}$, from which

(A17) $$\delta |\frac{dv(r)}{dr}{|}^{2}{\left(1+\delta |\frac{dv(r)}{dr}{|}^{2}\right)}^{-1}\leq 1.$$

Therefore, (A16) becomes

(A18) $$\begin{array}{c} F\left(r,u\left(r\right)-v\left(r\right),\frac{du(r)}{dr}-\frac{dv(r)}{dr}\right)<\\ <r^{-1}\left(\frac{du(r)}{dr}-\frac{dv(r)}{dr}\right)+4{\left(\theta \lambda f\left(r\right)\right)}^{-1}({\left(1-u\left(r\right)-d^{*}\right)}^{2}-\\ -{\left(1-v\left(r\right)-d^{*}\right)}^{2}+{\left(1-v\left(r\right)-d^{*}\right)}^{2}). \end{array}$$

Observing that ${\left(1-v\left(r\right)-d^{*}\right)}^{2}>0$, (A18) can be developed as follows:

(A19) $$\begin{array}{c} F\left(r,u\left(r\right)-v\left(r\right),\frac{du(r)}{dr}-\frac{dv(r)}{dr}\right)<\\ <r^{-1}\left(\frac{du(r)}{dr}-\frac{dv(r)}{dr}\right)+4{\left(\theta \lambda f\left(r\right)\right)}^{-1}\left({\left(1-u\left(r\right)-d^{*}\right)}^{2}+{\left(1-v\left(r\right)-d^{*}\right)}^{2}\right)<\\ <r^{-1}\left(\frac{du(r)}{dr}-\frac{dv(r)}{dr}\right)+4{\left(\theta \lambda f\left(r\right)\right)}^{-1}\left({\left(1-u\left(r\right)\right)}^{2}+{\left(1-v\left(r\right)\right)}^{2}\right)<\\ <r^{-1}\left(\frac{du(r)}{dr}-\frac{dv(r)}{dr}\right)+4{\left(\theta \lambda f\left(r\right)\right)}^{-1}\left(2+u^{2}\left(r\right)+v^{2}\left(r\right)-2u\left(r\right)-2v\left(r\right)\right)<\\ <r^{-1}\left(\frac{du(r)}{dr}-\frac{dv(r)}{dr}\right)+4{\left(\theta \lambda f\left(r\right)\right)}^{-1}\left(2+u\left(r\right)+v\left(r\right)-2u\left(r\right)-2v\left(r\right)\right)=\\ =r^{-1}\left(\frac{du(r)}{dr}-\frac{dv(r)}{dr}\right)+4{\left(\theta \lambda f\left(r\right)\right)}^{-1}\left(2-u\left(r\right)-v\left(r\right)\right)<\\ <r^{-1}\left(\frac{du(r)}{dr}-\frac{dv(r)}{dr}\right)+4{\left(\theta \lambda f\left(r\right)\right)}^{-1}\left(2+u\left(r\right)-v\left(r\right)\right)<\\ <r^{-1}\left(\frac{du(r)}{dr}-\frac{dv(r)}{dr}\right)+4{\left(\theta \lambda f\left(r\right)\right)}^{-1}\left(u\left(r\right)-v\left(r\right)\right), \end{array}$$

from which $L_{1}\left(r\right)=1/r$ and $K_{2}\left(r\right)=4/\left(\theta \lambda f\left(r\right)\right)$. Moreover, starting again from (20),

(A20) $$\begin{array}{c} -\frac{1}{\theta \lambda f(r)}=\left(\frac{d^{2}u\left(r\right)}{dr^{2}}+r^{-1}\frac{du(r)}{dr}\right)0.25{\left(1-u\left(r\right)-d^{*}\right)}^{-2}\left(1+\delta |\frac{du(r)}{dr}{|}^{2}\right)>\\ \left(\frac{d^{2}u\left(r\right)}{dr^{2}}+r^{-1}\frac{du(r)}{dr}\right)0.25{\left(1-u\left(r\right)-d^{*}\right)}^{-1}\left(1+\delta |\frac{du(r)}{dr}{|}^{2}\right)>\\ >0.25\left(\frac{d^{2}u\left(r\right)}{dr^{2}}+r^{-1}\frac{du(r)}{dr}\right){\left(1-u\left(r\right)-d^{*}\right)}^{-1}>0.25\left(\frac{d^{2}u\left(r\right)}{dr^{2}}+r^{-1}\frac{du(r)}{dr}\right). \end{array}$$

Similarly,

(A21) $$\begin{array}{c} -\theta \lambda f\left(r\right)>0.25\left(\frac{d^{2}v\left(r\right)}{dr^{2}}+r^{-1}\frac{dv(r)}{dr}\right), \end{array}$$

so that

(A22) $$\begin{array}{c} -2\theta \lambda f\left(r\right)>0.25\left(\frac{d^{2}u\left(r\right)}{dr^{2}}+r^{-1}\frac{du(r)}{dr}\right)+0.25\left(\frac{d^{2}v\left(r\right)}{dr^{2}}+r^{-1}\frac{dv(r)}{dr}\right)=\\ =0.25\left(\frac{d^{2}u\left(r\right)}{dr^{2}}+\frac{d^{2}v\left(r\right)}{d^{2}r}\right)+025r^{-1}\left(\frac{du(r)}{dr}+\frac{dv(r)}{dr}\right)>\\ >0.25r^{-1}\left(\frac{du(r)}{dr}+\frac{dv(r)}{dr}\right)>0.25r^{-1}\left(\frac{du(r)}{dr}-\frac{dv(r)}{dr}\right), \end{array}$$

from which

(A23) $$-8{\left(\theta \lambda f\left(r\right)\right)}^{-1}>0.25r^{-1}\left(\frac{du(r)}{dr}-\frac{dv(r)}{dr}\right).$$

Therefore,

(A24) $$\begin{array}{c} F(r,u\left(r\right)-v\left(r\right),\frac{du(r)}{dr}-\frac{dv(r)}{dr})=\\ r^{-1}\left(\frac{du(r)}{dr}-\frac{dv(r)}{dr}\right)+4{\left(1-u\left(r\right)-d^{*}\right)}^{2}{\left[\theta \lambda f\left(r\right)\left(1+\delta |\frac{du(r)}{dr}{|}^{2}\right)\right]}^{-1}-\\ -4{\left(1-v\left(r\right)-d^{*}\right)}^{2}{\left[\theta \lambda f\left(r\right)\left(1+\delta |\frac{dv(r)}{dr}{|}^{2}\right)\right]}^{-1}=\\ =r^{-1}\left(\frac{du(r)}{dr}-\frac{dv(r)}{dr}\right)+4{\left(\theta \lambda f\left(r\right)\right)}^{-1}({\left(1-u\left(r\right)-d^{*}\right)}^{2}{\left(1+\delta |\frac{du(r)}{dr}{|}^{2}\right)}^{-1}-\\ -{\left(1-v\left(r\right)-d^{*}\right)}^{2}{\left(1+\delta |\frac{dv(r)}{dr}{|}^{2}\right)}^{-1})\geq \\ \geq r^{-1}\left(\frac{du(r)}{dr}-\frac{dv(r)}{dr}\right)+4{\left(\theta \lambda f\left(r\right)\right)}^{-1}({\left(1-u\left(r\right)-d^{*}\right)}^{2}{\left(1+\delta |\frac{du(r)}{dr}{|}^{2}\right)}^{-1}-\\ -{\left(1-v\left(r\right)-d^{*}\right)}^{2}{\left(1+\delta |\frac{dv(r)}{dr}{|}^{2}\right)}^{-1})\geq \\ \geq r^{-1}\left(\frac{du(r)}{dr}-\frac{dv(r)}{dr}\right)+4{\left(\theta \lambda f\left(r\right)\right)}^{-1}({\left(1-u\left(r\right)-d^{*}\right)}^{2}{\left(1+\delta |\frac{du(r)}{dr}{|}^{2}\right)}^{-1}-\\ -{\left(1-v\left(r\right)-d^{*}\right)}^{2}{\left(1+\delta |\frac{dv(r)}{dr}{|}^{2}\right)}^{-1})=\\ =r^{-1}\left(\frac{du(r)}{dr}-\frac{dv(r)}{dr}\right)+\\ +4{\left(\theta \lambda f\left(r\right)\right)}^{-1}({\left(1+\delta |\frac{du(r)}{dr}{|}^{2}\right)}^{-1}{\left(1-u\left(r\right)-d^{*}\right)}^{2}\left(1+\delta \right|\frac{du(r)}{dr}{|}^{2}-\\ -\delta |\frac{du(r)}{dr}{|}^{2})-\left(1-v\left(r\right)-d^{*}\right))=\\ =r^{-1}\left(\frac{du(r)}{dr}-\frac{dv(r)}{dr}\right)+4{\left(\theta \lambda f\left(r\right)\right)}^{-1}({\left(1-u\left(r\right)-d^{*}\right)}^{2}-\\ -{\left(1+\delta |\frac{du(r)}{dr}{|}^{2}\right)}^{-1}{\left(1-u\left(r\right)-d^{*}\right)}^{2}\delta |\frac{du(r)}{dr}{|}^{2}-\left(1-v\left(r\right)-d^{*}\right))>\\ >r^{-1}\left(\frac{du(r)}{dr}-\frac{dv(r)}{dr}\right)+4{\left(\theta \lambda f\left(r\right)\right)}^{-1}({\left(1-u\left(r\right)-d^{*}\right)}^{2}-\\ -{\left(1-u\left(r\right)-d^{*}\right)}^{2}-\left(1-v\left(r\right)-d^{*}\right))=\\ =r^{-1}\left(\frac{du(r)}{dr}-\frac{dv(r)}{dr}\right)+4{\left(\theta \lambda f\left(r\right)\right)}^{-1}\left(-\left(1-v\left(r\right)-d^{*}\right)\right)>\\ >r^{-1}\left(\frac{du(r)}{dr}-\frac{dv(r)}{dr}\right)+4{\left(\theta \lambda f\left(r\right)\right)}^{-1}\left(-\left(1-v\left(r\right)-d^{*}\right)-\left(1-u\left(r\right)-d^{*}\right)\right)=\\ =r^{-1}\left(\frac{du(r)}{dr}-\frac{dv(r)}{dr}\right)+4{\left(\theta \lambda f\left(r\right)\right)}^{-1}\left(-1+v\left(r\right)+d^{*}-1+u\left(r\right)+d^{*}\right)=\\ =r^{-1}\left(\frac{du(r)}{dr}-\frac{dv(r)}{dr}\right)+4{\left(\theta \lambda f\left(r\right)\right)}^{-1}\left(-2+2d^{*}+u\left(r\right)+v\left(r\right)\right)\geq \\ \geq r^{-1}\left(\frac{du(r)}{dr}-\frac{dv(r)}{dr}\right)+4{\left(\theta \lambda f\left(r\right)\right)}^{-1}\left(-2+2d^{*}+u\left(r\right)-v\left(r\right)\right)>\\ >r^{-1}\left(\frac{du(r)}{dr}-\frac{dv(r)}{dr}\right)+4{\left(\theta \lambda f\left(r\right)\right)}^{-1}\left(-2+u\left(r\right)-v\left(r\right)\right)=\\ =r^{-1}\left(\frac{du(r)}{dr}-\frac{dv(r)}{dr}\right)-8{\left(\theta \lambda f\left(r\right)\right)}^{-1}\left(u\left(r\right)-v\left(r\right)\right). \end{array}$$

Finally, considering (A23), (A24) assumes the following form:

(A25) $$\begin{array}{c} F\left(r,u\left(r\right)-v\left(r\right),\frac{du(r)}{dr}-\frac{dv(r)}{dr}\right)>\\ >r^{-1}\left(\frac{du(r)}{dr}-\frac{dv(r)}{dr}\right)+0.5r^{-1}\left(\frac{du(r)}{dr}-\frac{dv(r)}{dr}\right)+{\left(\theta \lambda f\left(r\right)\right)}^{-1}\left(u\left(r\right)-v\left(r\right)\right)=\\ =1.25r^{-1}\left(\frac{du(r)}{dr}-\frac{dv(r)}{dr}\right)+{\left(\theta \lambda f\left(r\right)\right)}^{-1}\left(u\left(r\right)-v\left(r\right)\right). \end{array}$$

in which $L_{2}\left(r\right)=1.25r^{-1}$ and $K_{1}\left(r\right)={\left(\theta \lambda f\left(r\right)\right)}^{-1}$. To finally prove that $\frac{du_{1}\left(a\right)}{dr}\geq \frac{du_{2}\left(a\right)}{dr}$, we note that, since a=r=0, it follows that $\frac{du_{1}\left(a\right)}{dr}=\frac{d_{u_{1}\left(0\right)}}{dr}$. Furthermore, $\frac{du_{2}\left(a\right)}{dr}=\frac{du_{2}\left(0\right)}{dr}=0$, which completes the proof. □

## Appendix B

**Proof** **of** **Theorem** **2.** We start this proof by transforming model (20) into its equivalent integral formulation:

(A26) $$\begin{array}{c} u\left(r\right)=\int_{-R}^{R}G\left(r,s\right)(r^{-1}\frac{du(r)}{dr}+\\ +4{\left(\theta \lambda f\left(r\right)\right)}^{-1}{\left(1-u\left(r\right)-d^{*}\right)}^{2}{\left(1+\delta |\frac{du(r)}{dr}{|}^{2}\right)}^{-1})ds. \end{array}$$

where, for our problem, the Green’s function, G(r,s), is specified as follows:

(A27) $$G\left(r,s\right)=0.5R^{-1}\left(s+R\right)\left(R-r\right),\;\;\;\mathrm{if}\;\;-R\leq s\leq r,\;$$

(A28) $$G\left(r,s\right)=0.5R^{-1}\left(R-s\right)\left(r+R\right),\;\;\;\;\mathrm{if}\;\;r<s\leq R$$

Furthermore,

(A29) $$\frac{\partial G(r,s)}{\partial r}=-0.5R^{-1}\left(s+R\right),\;\;\;\mathrm{if}\;\;-R\leq s\leq r,$$

and

(A30) $$\frac{\partial G(r,s)}{\partial r}=0.5R^{-1}\left(R-s\right),\;\;\;\;\mathrm{if}\;\;r<s\leq R$$

Moreover,

(A31) 0≤G(r,s)≤0.5R∀r,s∈[−R,R]

and

(A32) $$\frac{\partial G(r,s)}{\partial r}\leq 0.5.$$

By contradiction, we consider two different solutions for (20), $u_{1}\left(r\right)$ and $u_{2}\left(r\right)$, such that $u_{1}\left(r\right)=T\left(u_{1}\left(r\right)\right)$ and $u_{2}\left(r\right)=T\left(u_{2}\left(r\right)\right)$. Therefore,

(A33) $$\begin{array}{c} u_{1}\left(r\right)=T\left(u_{1}\left(r\right)\right)=\int_{-R}^{R}G\left(r,s\right)(r^{-1}\frac{du_{1}\left(r\right)}{dr}+\\ +4{\left(\theta \lambda f\left(r\right)\right)}^{-1}{\left(1-u_{1}\left(r\right)-d^{*}\right)}^{2}{\left(1+\delta |\frac{du_{1}\left(r\right)}{dr}{|}^{2}\right)}^{-1})ds \end{array}$$

and

(A34) $$\begin{array}{c} u_{2}\left(r\right)=T\left(u_{2}\left(r\right)\right)=\int_{-R}^{R}G\left(r,s\right)(r^{-1}\frac{du_{2}\left(r\right)}{dr}+\\ +4{\left(\theta \lambda f\left(r\right)\right)}^{-1}{\left(1-u_{2}\left(r\right)-d^{*}\right)}^{2}{\left(1+\delta |\frac{du_{2}\left(r\right)}{dr}{|}^{2}\right)}^{-1})ds, \end{array}$$

from which

(A35) $$\begin{array}{c} u_{1}\left(r\right)=T\left(u_{1}\left(r\right)\right)=\int_{-R}^{R}\frac{\partial G(r,s)}{\partial r}(r^{-1}\frac{du_{1}\left(r\right)}{dr}+\\ +4{\left(\theta \lambda f\left(r\right)\right)}^{-1}{\left(1-u_{1}\left(r\right)-d^{*}\right)}^{2}{\left(1+\delta |\frac{du_{1}\left(r\right)}{dr}{|}^{2}\right)}^{-1})ds \end{array}$$

and

(A36) $$\begin{array}{c} u_{2}\left(r\right)=T\left(u_{2}\left(r\right)\right)=\int_{-R}^{R}\frac{\partial G(r,s)}{\partial r}(r^{-1}\frac{du_{2}\left(r\right)}{dr}+\\ +4{\left(\theta \lambda f\left(r\right)\right)}^{-1}{\left(1-u_{2}\left(r\right)-d^{*}\right)}^{2}{\left(1+\delta |\frac{du_{2}\left(r\right)}{dr}{|}^{2}\right)}^{-1})ds \end{array}$$

Therefore, taking into account also both (A31) and (A32), we can write:

(A37) $$\begin{array}{c} \left|\right|u_{1}\left(r\right)-u_{2}\left(r\right){\left|\right|}_{C^{1}\left(\left[-R,R\right]\right)}=\\ =\underset{r\in [-R,R]}{\sup}|u_{1}\left(r\right)-u_{2}\left(r\right)|+\underset{r\in [-R,R]}{\sup}\left|u_{1}^{\prime }\left(r\right)-u_{2}^{\prime }\left(r\right)\right|=\\ =\underset{r\in [-R,R]}{\sup}|\int_{-R}^{R}G\left(r,s\right)(r^{-1}\frac{du_{1}\left(r\right)}{dr}+\\ +4{\left(\theta \lambda f\left(r\right)\right)}^{-1}{\left(1-u_{1}\left(r\right)-d^{*}\right)}^{2}{\left(1+\delta |\frac{du_{1}\left(r\right)}{dr}{|}^{2}\right)}^{-1})ds-\\ -\int_{-R}^{R}G\left(r,s\right)(r^{-1}\frac{du_{2}\left(r\right)}{dr}+\\ +4{\left(\theta \lambda f\left(r\right)\right)}^{-1}{\left(1-u_{2}\left(r\right)-d^{*}\right)}^{2}{\left(1+\delta |\frac{du_{2}\left(r\right)}{dr}{|}^{2}\right)}^{-1})ds|+\\ +\underset{r\in [-R,R]}{\sup}|\int_{-R}^{R}\frac{\partial G(r,s)}{\partial r}(r^{-1}\frac{du_{1}\left(r\right)}{dr}+\\ +4{\left(\theta \lambda f\left(r\right)\right)}^{-1}{\left(1-u_{1}\left(r\right)-d^{*}\right)}^{2}{\left(1+\delta |\frac{du_{1}\left(r\right)}{dr}{|}^{2}\right)}^{-1})ds-\\ -\int_{-R}^{R}\frac{\partial G(r,s)}{\partial r}(r^{-1}\frac{du_{2}\left(r\right)}{dr}+\\ +4{\left(\theta \lambda f\left(r\right)\right)}^{-1}{\left(1-u_{2}\left(r\right)-d^{*}\right)}^{2}{\left(1+\delta |\frac{du_{2}\left(r\right)}{dr}{|}^{2}\right)}^{-1})ds|=\\ =\underset{r\in [-R,R]}{\sup}|\int_{-R}^{R}G\left(r,s\right)(r^{-1}\frac{du_{1}\left(r\right)}{dr}+\\ +4{\left(\theta \lambda f\left(r\right)\right)}^{-1}{\left(1-u_{1}\left(r\right)-d^{*}\right)}^{2}{\left(1+\delta |\frac{du_{1}\left(r\right)}{dr}{|}^{2}\right)}^{-1})-\\ -r^{-1}\frac{du_{2}\left(r\right)}{dr}+4{\left(\theta \lambda f\left(r\right)\right)}^{-1}{\left(1-u_{2}\left(r\right)-d^{*}\right)}^{2}{\left(1+\delta |\frac{du_{2}\left(r\right)}{dr}{|}^{2}\right)}^{-1})|+\\ +\underset{r\in [-R,R]}{\sup}|\int_{-R}^{R}\frac{\partial G(r,s)}{\partial r}(r^{-1}\frac{du_{2}\left(r\right)}{dr}+\\ +4{\left(\theta \lambda f\left(r\right)\right)}^{-1}{\left(1-u_{2}\left(r\right)-d^{*}\right)}^{2}{\left(1+\delta |\frac{du_{2}\left(r\right)}{dr}{|}^{2}\right)}^{-1})-\\ -r^{-1}\frac{du_{2}\left(r\right)}{dr}+4{\left(\theta \lambda f\left(r\right)\right)}^{-1}{\left(1-u_{2}\left(r\right)-d^{*}\right)}^{2}{\left(1+\delta |\frac{du_{2}\left(r\right)}{dr}{|}^{2}\right)}^{-1}))ds|\leq \\ \leq \underset{r\in [-R,R]}{\sup}\int_{-R}^{R}\left|G\left(r,s\right)\right||(r^{-1}\frac{du_{1}\left(r\right)}{dr}+\\ +4{\left(\theta \lambda f\left(r\right)\right)}^{-1}{\left(1-u_{1}\left(r\right)-d^{*}\right)}^{2}{\left(1+\delta |\frac{du_{1}\left(r\right)}{dr}{|}^{2}\right)}^{-1})-\\ -\left(r^{-1}\frac{du_{2}\left(r\right)}{dr}+4{\left(\theta \lambda f\left(r\right)\right)}^{-1}{\left(1-u_{2}\left(r\right)-d^{*}\right)}^{2}{\left(1+\delta |\frac{du_{2}\left(r\right)}{dr}{|}^{2}\right)}^{-1}\right)|ds+\\ +\underset{r\in [-R,R]}{\sup}\int_{-R}^{R}|\frac{\partial G(r,s)}{\partial r}||(r^{-1}\frac{du_{1}\left(r\right)}{dr}+4{\left(\theta \lambda f\left(r\right)\right)}^{-1}{\left(1-u_{1}\left(r\right)-d^{*}\right)}^{2}\\ {\left(1+\delta |\frac{du_{1}\left(r\right)}{dr}{|}^{2}\right)}^{-1}))-\\ -\left(r^{-1}\frac{du_{2}\left(r\right)}{dr}+4{\left(\theta \lambda f\left(r\right)\right)}^{-1}{\left(1-u_{2}\left(r\right)-d^{*}\right)}^{2}{\left(1+\delta |\frac{du_{2}\left(r\right)}{dr}{|}^{2}\right)}^{-1}\right)|ds\leq \\ \leq 0.5R\underset{r\in [-R,R]}{\sup}\int_{-R}^{R}|r^{-1}\frac{du_{1}\left(r\right)}{dr}-r^{-1}\frac{du_{2}\left(r\right)}{dr}+\\ +4{\left(\theta \lambda f\left(r\right)\right)}^{-1}\left(4{\left(\theta \lambda f\left(r\right)\right)}^{-1}{\left(1-u_{1}\left(r\right)-d^{*}\right)}^{2}{\left(1+\delta |\frac{du_{1}\left(r\right)}{dr}{|}^{2}\right)}^{-1}\right)-\\ -4{\left(\theta \lambda f\left(r\right)\right)}^{-1}{\left(1-u_{2}\left(r\right)-d^{*}\right)}^{2}{\left(1+\delta |\frac{du_{2}\left(r\right)}{dr}{|}^{2}\right)}^{-1})|ds+\\ +\frac{1}{2}\underset{r\in [-R,R]}{\sup}\int_{-R}^{R}|r^{-1}\frac{du_{1}\left(r\right)}{dr}-r^{-1}\frac{du_{2}\left(r\right)}{dr}+\\ +4{\left(\theta \lambda f\left(r\right)\right)}^{-1}\left(4{\left(\theta \lambda f\left(r\right)\right)}^{-1}{\left(1-u_{1}\left(r\right)-d^{*}\right)}^{2}{\left(1+\delta |\frac{du_{1}\left(r\right)}{dr}{|}^{2}\right)}^{-1}\right)-\\ -4{\left(\theta \lambda f\left(r\right)\right)}^{-1}{\left(1-u_{2}\left(r\right)-d^{*}\right)}^{2}{\left(1+\delta |\frac{du_{2}\left(r\right)}{dr}{|}^{2}\right)}^{-1})|ds=\\ =0.5\left(1+R\right)\underset{r\in [-R,R]}{\sup}\int_{-R}^{R}|r^{-1}\frac{du_{1}\left(r\right)}{dr}-r^{-1}\frac{du_{2}\left(r\right)}{dr}+\\ +4{\left(\theta \lambda f\left(r\right)\right)}^{-1}\left(4{\left(\theta \lambda f\left(r\right)\right)}^{-1}{\left(1-u_{1}\left(r\right)-d^{*}\right)}^{2}{\left(1+\delta |\frac{du_{1}\left(r\right)}{dr}{|}^{2}\right)}^{-1}\right)-\\ -4{\left(\theta \lambda f\left(r\right)\right)}^{-1}{\left(1-u_{2}\left(r\right)-d^{*}\right)}^{2}{\left(1+\delta |\frac{du_{2}\left(r\right)}{dr}{|}^{2}\right)}^{-1})|ds. \end{array}$$

Observing that

(A38) $$\begin{array}{c} {\left(1+\delta |\frac{du(r)}{dr}{|}^{2}\right)}^{-1}={\left(\theta \lambda f\left(r\right)\right)}^{-1}\left(1+\delta |\frac{du(r)}{dr}{|}^{2}-\delta |\frac{du(r)}{dr}{|}^{2}\right)\leq \\ \leq 1+\delta |\frac{du(r)}{dr}{|}^{2}-\delta |\frac{du(r)}{dr}{|}^{2}\leq 1+\delta |\frac{du(r)}{dr}{|}^{2}<1+\delta H^{2}, \end{array}$$

inequality (A37) becomes:

(A39) $$\begin{array}{c} \left|\right|u_{1}\left(r\right)-u_{2}\left(r\right){\left|\right|}_{C^{1}\left(\left[-R,R\right]\right)}\leq \\ \leq 0.5\left(1+R\right)\underset{r\in [-R,R]}{\sup}\{\int_{-R}^{R}r^{-1}|\frac{du_{1}\left(r\right)}{dr}-\frac{du_{2}\left(r\right)}{dr}|ds+\\ +4{\left(\theta \lambda f\left(r\right)\right)}^{-1}\int_{-R}^{R}{\left(1-u_{1}\left(r\right)-d^{*}\right)}^{2}{\left(1+\delta H^{2}\right)}^{2}ds+\\ 4{\left(\theta \lambda f\left(r\right)\right)}^{-1}\int_{-R}^{R}{\left(1-u_{2}\left(r\right)-d^{*}\right)}^{2}\left(1+\delta H^{2}\right)ds\}ds\leq \\ \leq 0.5\left(1+R\right)\underset{r\in [-R,R]}{\sup}\{\int_{-R}^{R}r^{-1}\left|u_{1}^{\prime }\left(r\right)-u_{2}^{\prime }\left(r\right)\right|ds+\\ +4{\left(\theta \lambda f\left(r\right)\right)}^{-1}{\left(1+\delta H^{2}\right)}^{2}\int_{-R}^{R}\left[{\left(1-u_{1}\left(r\right)\right)}^{2}+{\left(1-u_{2}\left(r\right)\right)}^{2}\right]ds\}=\\ =0.5\left(1+R\right)\underset{r\in [-R,R]}{\sup}\{2Rr^{-1}|\frac{du_{1}\left(r\right)}{dr}-\frac{du_{2}\left(r\right)}{dr}|+\\ +8R{\left(\theta \lambda f\left(r\right)\right)}^{-1}\left(1+\delta H^{2}\right)\left[{\left(1-u_{1}\left(r\right)\right)}^{2}+{\left(1-u_{2}\left(r\right)\right)}^{2}\right]ds\}=\\ =0.5\left(1+R\right)2Rr^{-1}\underset{r\in [-R,R]}{\sup}|\frac{du_{1}\left(r\right)}{dr}-\frac{du_{2}\left(r\right)}{dr}|+\\ +4\left(1+R\right)R{\left(\theta \lambda f\left(r\right)\right)}^{-1}\left(1+\delta H^{2}\right)\underset{r\in [-R,R]}{\sup}\left[{\left(1-u_{1}\left(r\right)\right)}^{2}+{\left(1-u_{2}\left(r\right)\right)}^{2}\right]\leq \\ \leq 2\left(R+1\right)R\underset{r\in [-R,R]}{\sup}|\frac{du_{1}\left(r\right)}{dr}-\frac{du_{2}\left(r\right)}{dr}|+\\ +{\left(\theta \lambda f\left(r\right)\right)}^{-1}\left(4R+4R^{2}\right)\left(1+\delta H^{2}\right)\underset{r\in [-R,R]}{\sup}\left|u_{2}\left(r\right)-u_{1}\left(r\right)\right|. \end{array}$$

For achieving a contradiction, from (A39), it is necessary that

(A40) 2(R+1)R<1

and

(A41) $${\left(\theta \lambda f\left(r\right)\right)}^{-1}\left(4R+4R^{2}\right)\left(1+\delta H^{2}\right)<1.$$

If it were

(A42) $${\left(\theta \lambda f\left(r\right)\right)}^{-1}\left(4R+4R^{2}\right)\left(1+\delta H^{2}\right)<2\left(R+1\right)R,$$

the following inequality would hold:

(A43) $$H<{\left(\left(0.5\theta \lambda f\left(r\right)-1\right)\delta^{-1}\right)}^{1/2}.$$

However, (A43) is false because, without a fringing field, it would cause H→+∞ with consequent adherence of the membrane along the walls of the micropump (i.e., $\max\{|u^{\prime }\left(r\right)|\}$ would correspond to r=±R). However, it was proved in [50] that $\max\{|u^{\prime }\left(r\right)|\}<\sup\{|u^{\prime }\left(r\right)\left|\right\}=H=146$, so that, on x=±R, there is no danger of adhesion between the membrane and the walls of the micropump, which occurs when $|u^{\prime }\left(\pm R\right)|\to +\infty$. Therefore, the uniqueness of the solution for (20) is ensured if (31) yields. □
